# DNA minor-groove binder Hoechst 33258 destabilizes base-pairing adjacent to its binding site

**DOI:** 10.1038/s42003-020-01241-4

**Published:** 2020-09-22

**Authors:** Xin-Xing Zhang, Shelby L. Brantley, Steven A. Corcelli, Andrei Tokmakoff

**Affiliations:** 1 Department of Chemistry, James Franck Institute, and Institute for Biophysical Dynamics, University of Chicago, 929 E. 57th St., Chicago, IL 60637 USA; 2 Department of Chemistry and Biochemistry, University of Norte Dame, Notre Dame, IN 46556 USA

**Keywords:** Biophysical chemistry, DNA

## Abstract

Understanding the dynamic interactions of ligands to DNA is important in DNA-based nanotechnologies. By structurally tracking the dissociation of Hoechst 33258-bound DNA (d(CGCAAATTTGCG)_2_) complex (*H-DNA*) with T-jump 2D-IR spectroscopy, the ligand is found to strongly disturb the stability of the three C:G base pairs adjacent to A:T the binding site, with the broken base pairs being more than triple at 100 ns. The strong stabilization effect of the ligand on DNA duplex makes this observation quite striking, which dramatically increases the melting temperature and dissociation time. MD simulations demonstrate an important role of hydration water and counter cations in maintaining the separation of terminal base pairs. The hydrogen bonds between the ligand and thymine carbonyls are crucial in stabilizing *H-DNA*, whose breaking signal appearing prior to the complete dissociation. Thermodynamic analysis informs us that *H-DNA* association is a concerted process, where *H* cooperates with DNA single strands in forming *H-DNA*.

## Abstract (web summary)

Using T-jump 2D-IR spectroscopy, Zhang et al. show that DNA minor-groove binder Hoechst 33258 strongly destabilizes its complex with DNA (H-DNA). This study suggests that the association of H-DNA is a concerted process, where Hoechst 33258 cooperates with DNA single strands in forming H-DNA.

## Introduction

The dye Hoechst 33258 (H) and its derivatives are widely used as fluorescent cytological stains for regulating gene expression^1–6^, detecting the local environment and structural vibrations of DNA^7–11^, because of their sequence-specific binding with DNA^12–18^. The association of H with DNA duplex (dsDNA), is entropy-driven and proceeds with a large and unfavorable change in enthalpy^19^. The hydration entropy change, resulting from the release of water in the DNA minor groove to the bulk, is much larger than the change in configurational entropy^20–26^. The displacement of hydration water by H highly improves the stability of dsDNA structure with subtle conformational distortions^27–29^. But due to the lack of techniques with sufficient structural sensitivity and time resolution, little effort^30,31^ has been spent on the dynamic impact of ligands on the association dynamics and structural vibrations of ligand-bound DNA complex.

To solve the experimental challenges in the characterization of the small structural variations of DNA occurring during the multistep association/dissociation process, we introduce here the temperature jump (T-jump) two-dimensional infrared (2D-IR) spectroscopy, which tracks the changes in DNA configuration and its hydrogen-bond networks from nanoseconds to milliseconds, by monitoring the unique fingerprint region of nucleobases and their vibrational couplings^32^. The DNA dodecamer d(CGCAAATTTGCG)_2_ is employed for the good stability of terminal base pairs and its unique binding mode to H with 1:1 stoichiometry at physiological pH^33,34^. Our data reveal that, in the association of H-DNA*,* H works concertedly with DNA single strands (ssDNA) rather than directly binding to the specific site on dsDNA. The H binding can strongly disturb the stability of the three C:G base pairs adjacent to A:T the binding site, even though the DNA structure varies only slightly. Unlike the fraying-peeling mechanism in native DNA^35^, the dissociation of central A:T pairs in H-DNA is initiated by breaking the hydrogen bonds between H and thymine carbonyls rather than the fraying of terminal base pairs. Such dynamical distributions can be a concern in the applications of small ligands as sensors detecting the local environment of bimolecular.

## Results

### Thermal dissociation of native and H-DNA

**Fig. 1:**
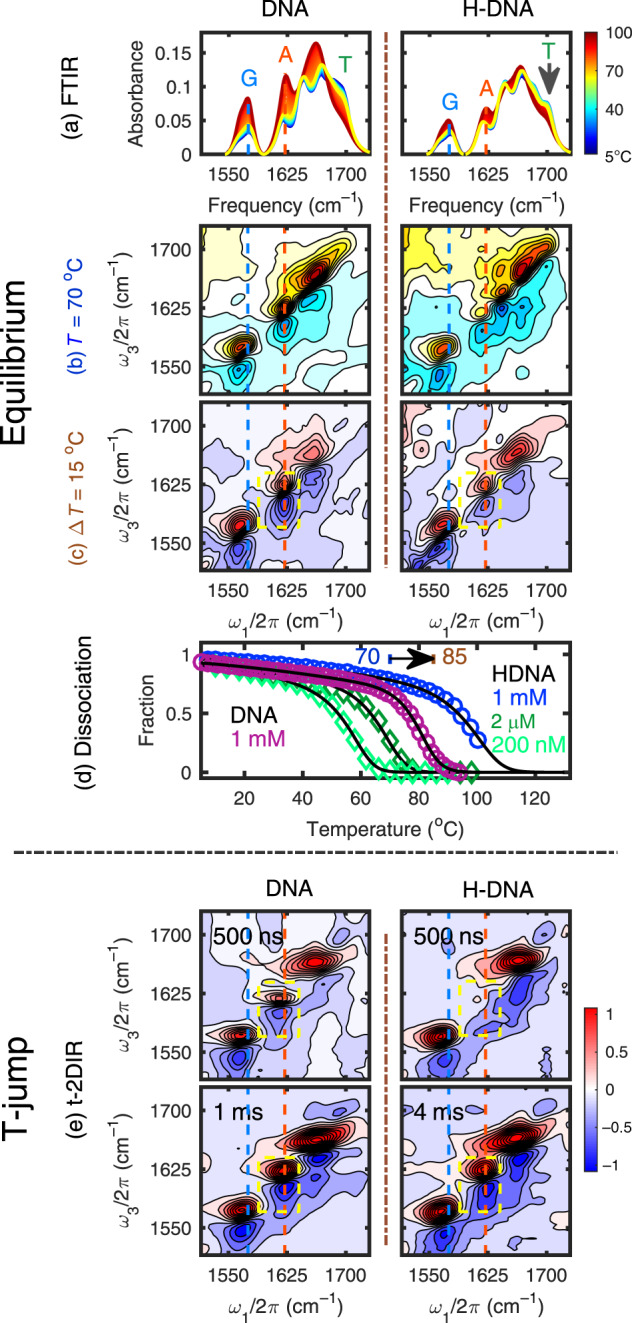
Infrared spectra of 1 mM DNA (left) and H-DNA (right). **a** Temperature-ramp FTIR spectra from 5 to 100 °C. **b** 2D-IR surfaces of DNA and H-DNA measured at 70 °C with parallel polarization. **c** The difference spectra between 85 and 70 °C surfaces. **d** Melting curve obtained with SVD analysis of the FTIR spectra for 1 mM DNA and 1 mM H-DNA (circles) and normalized temperature-dependent fluorescence intensity at 446 nm for 200 nM and 2 μM H-DNA (diamonds). The fitted curves obtained from a two-state model are shown with black solid lines. **e** Transient 2D-IR spectra of native DNA at 500 ns and 1 ms and H-DNA spectra at 500 ns and 4 ms, measured at *T*_i_ = 70 °C and *T*_f_ = 85 °C with parallel polarization. The spectral feature associated with the loss of A:T base pairs is outlined with a yellow box.

The binding of H to the DNA minor groove stabilizes the duplex and leads to structural changes caused by the formation of hydrogen bonds with the ligand and the loss of hydration^26,36^. As an initial assessment, we performed temperature-dependent FTIR measurements between 5 and 100 °C to track the DNA double strand (ds) to single strand (ss) melting transition (Fig. 1a). At high temperatures, the ssDNA IR spectrum does not differ much from the composition weighted sum of the individual free nucleotides^32^. At low temperatures for the duplex DNA, the coupling of vibrational modes between bases that result from hydrogen-bonding and stacking interactions strongly influence their frequencies, intensities, and line shapes^32^. The dominant feature of A:T base-pair dissociation is the dramatic intensity gain of the A ring mode at 1622 cm^−1^, which is suppressed in the stacking of hydrogen-bonded Watson-Crick pairs. Similarly, the G ring modes at 1564 and 1575 cm^−1^ are likewise suppressed in a duplex structure. These features report on the status of G:C pairs, although they overlap with the A ring mode at 1574 cm^−1^ whose intensity varies slightly with rising temperature (Supplementary Fig. 1).

For the specific characterization of A:T or G:C base pairs, 2D-IR spectroscopy is employed to provide additional structural information through the lineshape of peaks and the crosspeaks between different resonances^37^. Comparing the spectra for DNA and H-DNA at 70 °C (Fig. 1b), the most apparent spectral differences are observed in the 1610–1660 cm^−1^ range and near 1700 cm^−1^, which reports on ligand-induced thermal stability and structural variations of DNA duplexes. The most remarkable intensity differences are observed at the diagonal peak 1622 cm^−1^ and the cross peak at *ω*_1_/*ω*_3_ = 1677/1639 cm^−1^, indicating the relatively unstable structure of native DNA duplexes. Furthermore, the diagonal peak at 1702 cm^−1^ of H-DNA is clearly stronger than that of native DNA, appearing with a cross peak *ω*_1_/*ω*_3_ = 1670/1702 cm^−1^, arising from the thymine (T) C^2^=O carbonyl. These changes are similar to those observed by Hunt and co-workers for H binding to duplex 5′-GCAAATTTGC-3′ ^26^. Such behavior has also been observed in the FTIR spectra, a shoulder peak appears at 1702 cm^−1^ upon ligand binding (highlighted with an arrow in Fig. 1a), which originates from the T carbonyl stretching mode at 1698 cm^−1^ (Supplementary Figs. 2 and 3). Other than the T carbonyl feature, the temperature difference spectra of native and H-DNA are similar (Fig. 1c), consisting of three dominant doublet contours on the diagonal representing intensity gain upon DNA melting. Both the A ring mode at 1622 cm^−1^ and the G ring modes at 1540–1580 cm^−1^ increase in intensity with the loss of base-pair stacking.

The IR spectra of H-DNA show much smaller changes compared to native DNA over the same temperature range, indicating a strong stabilization effect to the duplex upon H binding^30,31^. To describe the global spectral changes that occur with thermal dissociation, we analyzed the temperature-dependent IR spectra using single value decomposition (SVD). Since the peak intensity of a free ligand *H* is ~100 times smaller than the DNA and insensitive to temperature, the melting curves obtained from the normalized second SVD component report primarily on the dsDNA-to-ssDNA transition (Fig. 1d). Due to sensitivity limitations, a concentrated 1 mM H-DNA solution with 1:1 H-to-dsDNA stoichiometry was used in the IR measurements; however, the melting transition for H-DNA extends above 100 °C at this concentration. To characterize the complete thermal melting of H-DNA, fluorescence spectroscopy is employed for studies at lower concentrations. Upon binding to the duplex minor groove, H is stabilized in a rigid form resulting in an increase in fluorescence at 446 nm (Supplementary Fig. 4), which can be used to characterize the concentration of H-DNA. The normalized temperature-dependent intensity changes of 2 µM and 200 nM H-DNA are shown in Fig. 1d with color-coded diamonds. The melting curves of H-DNA show clear concentration dependence, with their inflection point ranging from ~50 to ~100 °C.

### Dissociation of H-DNA at equilibrium

The melting curves obtained with FTIR or fluorescence spectroscopy are influenced by the dsDNA–ssDNA melting transition and the fraying of base pairs within a duplex. IR spectra are sensitive to hydrogen-bond breaking and stacking fluctuations that occur during fraying or bubbling, whereas the fluorescence intensity is correlated with changes in structural rigidity of H resulting from DNA conformational changes. As a result, the melting curves observed by IR and fluorescence measurements (Fig. 1d) report on the fraction of intact base pairs *θ* (Supplementary Eq. S2). The dissociation of native DNA is a simple two-state transition (D ⇄ 2S) involving the dissociation of the duplex (D) into two single strands (S), which can be described with a dissociation constant *K*_d_. In contrast, the H-DNA dissociation is a more complicated process mechanistically due to the three binding partners involved, two S and one ligand (H).

The binding thermodynamics of H-DNA is discussed in detail in the Supplementary Section 4. Briefly, there are three possible schemes by which to analyze H-DNA melting curves. Since H does not bind to ssDNA in pH 7 buffer due to the presence of counter cations (Na^+^) that disrupt the nonspecific electronic interactions, we can neglect schemes with S and H bound^13^. Using a three-state model for stepwise hybridization and binding (2S ⇄ D, D + H ⇄ DH), we find that binding constant *K*_b_ of H with D analyzed is strongly dependent on the concentration of DNA strands and much smaller than the value determined by fluorescence^12^, indicating problems with this model. Instead we find that the dissociation of H-DNA is better characterized as a two-state concerted dissociation into its three constituents (DH ⇄ H + 2S) in this range, using a Gibbs free energy change for the dissociation process,

1 $${\Delta}G_{\mathrm{d}}^0\left( T \right) = {\Delta}H_{\mathrm{d}}^0 - T{\Delta}S_{\mathrm{d}}^0 = - RT\ln (K_{\mathrm{d}})$$

The results for modeling the DNA melting curves with and without the presence of H are summarized in Table 1 with the detailed fitting process discussed in Supplementary Section 5. To simplify the self-consistent description of all data we use a reference temperature *T*_0_, defined as the temperature at which ${\Delta}G_{\mathrm{d}}^0 = 0$, instead of the traditional concentration-dependent melting temperature *T*_m_ at which the duplex fraction is 0.5. *T*_0_ is concentration independent for H-DNA dissociation and 45 °C higher than that of native DNA, indicating a strong stabilization effect of ligand binding. Note, in the temperature range of 85–100 °C and at 1 mM concentration, conditions exist where the duplex DNA is unstable without H bound. The enthalpy change Δ*H*_d_ of H-DNA dissociation reflects the internal energy changes resulting from the disruption of noncovalent interactions between the three binding partners and solvent reorganization, specifically near the binding interface, and has a somewhat larger value than that of native DNA. In contrast, the entropy change Δ*S*_d_ of H-DNA is ~9% smaller than that of native DNA, arising mainly from a large solvent entropy change due to the release of water molecules to the bulk^23,24^. The intrinsic configurational entropy and contributions from the decrease in translational/rotational degrees of freedom are insignificant in comparison^23^.

**Table 1:** Thermodynamics parameters for melting transition.

Sample	*C*_D_ (M)	*T*_m_ (^o^C)^a^	*T*_0_ (^o^C)^b^	${\Delta}H_{\mathrm{d}}^0$ (kJ mol^−1^)	${\Delta}S_{\mathrm{d}}^0$ (J mol^−1^ K^−1^)
DNA	1 × 10^−3^	77	97	403	1088
H-DNA	1 × 10^−3^	93	142	415	1000
	2 × 10^−6^	63			
	2 × 10^−7^	52			

^a^*T*_m_ is defined as the temperature at which the DNA duplex fraction is 0.5.

^b^*T*_0_ is defined as the temperature where *K*_d_ = 1 M and ${\Delta}G_{\mathrm{d}}^0$ = 0.

### Dissociation of H-DNA observed by T-jump IR

To further investigate the effect of ligand binding on the duplex dissociation pathway, we tracked the change of IR spectra over seven decades in time in response to a nanosecond T-jump. Transient 2D-IR measurements shown in Fig. 1e show the spectral changes at time delays of 500 ns and 4 ms following a 15 °C T-jump from an initial temperature *T*_i_ = 70 °C. In this example, the time delays are selected to be much shorter and approximately equal to the timescale for duplex dissociation, and using equal temperatures allows one to properly compare dissociation kinetics. The millisecond spectra for H-DNA are similar to the spectral changes observed for the same temperature range under equilibrium conditions (Fig. 1c), indicating that dissociation of the duplexes is nearly complete by this time. In contrast, the 500 ns transient 2D-IR spectra of DNA and H-DNA are clearly different in the 1600–1650 cm^−1^ range, corresponding to the A ring vibration. The DNA spectrum at 500 ns shares similar spectral changes to the peaks at longer delays reporting on fast disruption of G:C and A:T contacts, although with a much reduced amplitude. In contrast, the 500 ns spectrum for H-DNA shows no changes to the A:T features and a much-enhanced signal change for the G:C peak, revealing that the fast response of H-DNA originates only from the G:C base pairs. By calculating the integrated intensity of G:C and A:T spectral features displayed in Fig. 1e, we find that 62% G:C and 32% A:T base pairs in the native DNA duplex break by 500 ns, prior to fully dissociating at 1 ms. With H bound, all G:C base pairs have dissociated by 500 ns but no disruption of A:T pairing is distinguishable. These observations illustrate how binding of H to the central AAATTT tracts stabilizes the duplex core to dissociation, but at the cost of destabilizing and rapidly fraying the G:C termini.

**Fig. 2:**
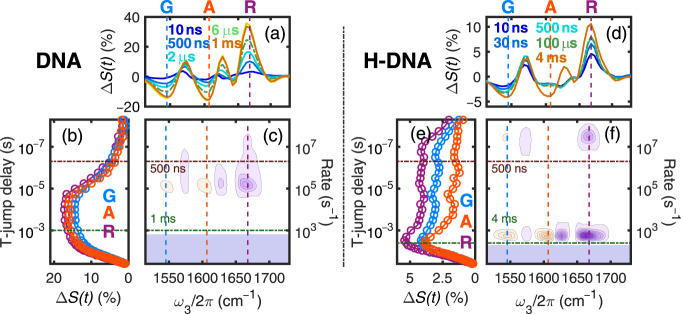
Dissociation kinetics of DNA and H-DNA observed with T-jump experiments measured at *T*_i_ = 70 °C and *T*_f_ = 85 °C with parallel polarization. **a**, **d** Representative t-HDVE spectra at specified delays. **b**, **e** Single-frequency kinetic traces on a log timescale representing the dissociation of A:T (A = 1607 cm^−1^), G:C (G = 1546 cm^−1^), and all base pairs (R = 1668 cm^−1^). The amplitude of the R traces is rescaled by a factor of 0.5. Frequency slices are indicated by the color-coded lines in (**c**) and (**f**). **c**, **f** Rate distributions of the T-jump data. The positive and negative amplitudes are shown in orange and purple, respectively. Shaded areas indicate the relaxation kinetics concurrent with the temperature re-equilibration. The T-jump signal change is rescaled by the maximum spectral intensity at equilibrium described by ${\Delta}S\prime (t) = {\Delta}S(t)/\max \left( {S_{{\mathrm{eq}}}(T_{\mathrm{i}})} \right)$.

To monitor the kinetics at finer time steps, transient heterodyned dispersed vibrational echo (t-HDVE) spectra were recorded for delays between 10 ns and 50 ms instead of the full 2D-IR surfaces to shorten data acquisition time. These spectra provide a projection of the 2D-IR spectra onto the detection axis *ω*_3_. By tracking changes to the G ring mode (1546 cm^−1^) and the A ring mode (1607 cm^−1^), spectra can independently track dissociation of G:C and A:T pairs. The T-jump spectral evolution of DNA and H-DNA as a function of time delay are distinct at 1607 and 1668 cm^−1^ (Fig. 2a, d). These traces show the fractional signal change relative to the signal change under equilibrium conditions for time delays between 10 ns and 50 ms. Due to the temperature jump, the increased thermal fluctuations in the solvent and DNA give rise to the signal change in the t-HDVE spectrum at 10 ns. At 70 °C, the maximum spectral intensities of native and H-DNA are nearly equal because both are predominately in the dimer form. In general, the T-jump response of DNA in the absence of H is larger than that of H-DNA, consistent with the changes in duplex fraction expected from the melting curves across this 70–85 °C temperature range (Fig. 1d). For H-DNA, no clear intensity changes at 1607 cm^−1^ are observed up to 100 µs, consistent with the ligand binding strongly stabilizing the A:T base pairs (Fig. 2e). In contrast, within the first 100 ns after the T-jump, signal changes for H-DNA at 1668 cm^−1^ rise to 66% of its maximum (Fig. 2a trace R) instead of 17% of native DNA (Fig. 2b trace R), suggesting a destabilization effect on the G:C termini. The G:C kinetic trace for H-DNA is distinct from the A:T trace showing bimodal kinetics (Fig. 2e), whereas the representative kinetic traces of native DNA are nearly identical (Fig. 2b).

To provide a model free summary of the complex relaxation processes observed in these experiments, a numerical inverse Laplace transform using the maximum entropy method (MEM) is applied to the T-jump data to illustrate the distribution of observed relaxation rates *λ*_obs_ associated with each spectral feature^38,39^. The representative MEM distribution of *λ*_obs_ for DNA and H-DNA are shown for relaxation rates between 10^8^ and 10^1^ s^−1^ (Fig. 2c, f). The orange and purple contours correspond to positive and negative changes in the spectral amplitudes, respectively. Features with rates below 4 × 10^2^ s^−1^ in the shaded region arise from the thermal re-equilibration of the sample during cooling. Dashed lines serve as frequency markers for the dissociation of A:T and G:C pairs and are color-coded according to the time traces in Fig. 2b, e. In the presence of H, two negative features centered at 1570 and 1665 cm^−1^ are observed at *λ*_obs_ = 3 × 10^7^ s^−1^ (Fig. 2f), which are assigned to the change to the G ring and carbonyl mode upon the loss of G:C pairs, respectively. The amplitude of these features decreases consistently with rising temperature, however, their average rate does show clear temperature dependence, only varying by about 3 × 10^7^ s^−1^ and impacted by the 8 ns T-jump pulse (Supplementary Fig. 9).

The dsDNA-to-ssDNA transition can be characterized by the dissociation of A:T pairs, which is observed as paired positive/negative features centered at 1606/1627 cm^−1^ in the rate distribution plot. Note that the spectral features reflecting the loss of G:C pairs are also observed in the same timescale, indicating that the remaining intact G:C and A:T base pairs dissociate concertedly. The average observed rate is ~10^5^ s^−1^ for native DNA but is slowed dramatically down to ~2 × 10^3^ s^−1^ in the presence of H.

**Fig. 3:**
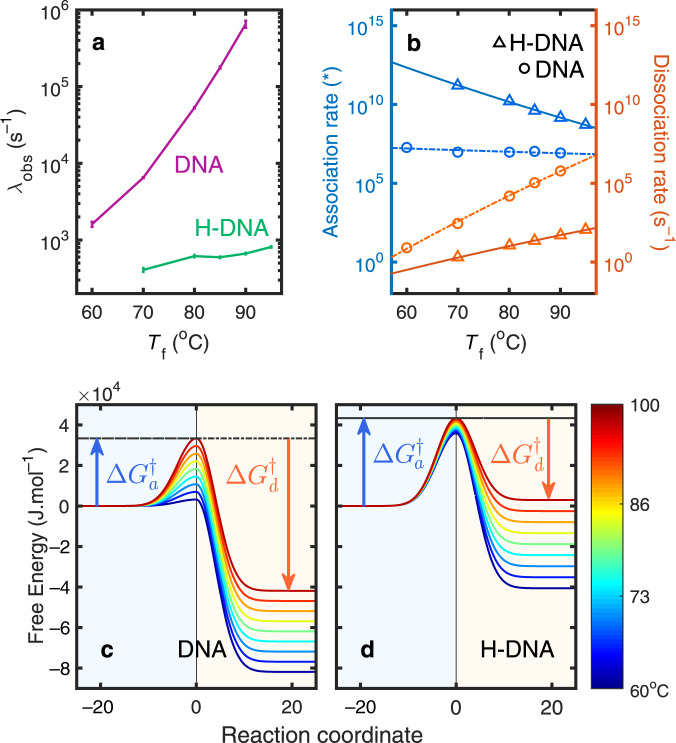
Transition rate and free energy barriers. **a** Average observed rate (*λ*_obs_) calculated from the amplitude-weighted mean across the maximum. Error bars represent the standard deviation of the averaged rates. **b** Calculated association (*k*_a_) and dissociation (*k*_d_) rate constants of DNA (circles) and H-DNA (triangles) and their fits from Eq. (2). *The unit of *k*_a_ is M^−1^ s^−1^ in the DNA association, and M^−2 ^s^−1^ in the H-DNA association. **c**, **d** Free energy barriers of association (${\Delta}G_{\mathrm{a}}^\dagger$) and dissociation (${\Delta}G_{\mathrm{d}}^\dagger$) of DNA and H-DNA.

To quantify activation energies for these processes, T-jump measurements with *T*_f_ varying between 60 and 95 °C and fixed Δ*T* = 15 °C were acquired (Fig. 3a and Supplementary Table S4). These temperatures lie in a regime where the duplex in the absence of H are destabilized. *λ*_obs_ of H-DNA varies slightly with the *T*_f_ in the range of 410 (*T*_f_ = 70 °C) to 810 s^−1^ (*T*_f_ = 95 °C), whereas that of native DNA rises steeply with temperature from 1.6 × 10^3^ s^−1^ (*T*_f_ = 60 °C) to 6.5 × 10^5^ s^−1^ (*T*_f_ = 90 °C). To better compare and quantify the rates for native and H-DNA dissociation, the amplitude-weighted mean of rates across all frequencies was calculated. To avoid the inaccuracy caused by spectral noise, only the rate distributions with amplitude above half maximum are included in the calculation for each frequency.

### Dissociation kinetics of H-DNA

As discussed earlier, the dissociation of H-DNA can be complicated by passing through various possible metastable intermediates, such as the H-free duplex, D, or the H bound to single strands, HS. Since the dsDNA oligonucleotide is not stable at the temperatures we investigated, D will not exist as a stable intermediate state in the H-DNA dissociation scheme. Thus, we describe the dissociation kinetics of both native DNA and H-DNA with the same two-state model as above, DH ⇄ H + 2S. To separate the dissociation (*k*_d_) and association (*k*_a_) rate constants for this reaction, we make use of the equilibrium constant, *K*_d_ = *k*_d_/*k*_a_, using Eq. (1) with the parameters in Table 1, and the relaxation rate for this process derived in the Supplementary Eq. (S23): $\lambda _{{\mathrm{obs}}} = 3\left[ S \right]_{{\mathrm{eq}}}^2k_{\mathrm{a}} + k_{\mathrm{d}}$, where [S]_eq_ is expressed with *K*_d_ and *C*_D_ in the Supplementary Eq. (S16). To quantify the barriers for the dissociation and association reactions, we analyzed the temperature-dependent kinetics using the Eyring equation

2 $${\Delta}G_{\mathrm{d}}^0 = RT\left[ {\ln \left( {k_{\mathrm{a}}} \right) - \ln \left( {k_{\mathrm{d}}} \right)} \right] = {\Delta}G_{\mathrm{d}}^\dagger - {\Delta}G_{\mathrm{a}}^\dagger$$

3 $$k_i = k_0\exp \left[ {\frac{{ - {\Delta}G_i^\dagger }}{{RT}}} \right] = k_0\exp \left[ {\frac{{{\Delta}S_i^\dagger }}{R} - \frac{{{\Delta}H_i^\dagger }}{{RT}}} \right],$$

where *i* = *a* or *d* for the association and dissociation process, respectively. The attempt frequency *k*_0_ equals *k*_B_T/*h*, where *h* is Planck’s constant and *k*_B_ is Boltzmann constant. In evaluating the rate expressions, we assumed that the final temperature of the T-jump, *T*_f_, was the operational temperature for the kinetics.

The results of the kinetic modeling are presented in Table 2. For the association process, both the activation enthalpy and entropy of H-DNA are one order of magnitude higher than that of native DNA. The variation in ${\Delta}G_a^\dagger$ with increasing temperature is very subtle (Fig. 3c), while H-DNA has more dramatic variation resulting from the cooperative hybridization/binding process (Fig. 3d). Similarly, the dissociation process is affected strongly by the ligand binding (which happens only above 70 °C, Fig. 3d). Besides the lesser configurational flexibility of H-DNA, replacement of H with water molecules also contributes to the entropic change, resulting in a ${\Delta}S_{\mathrm{d}}^\dagger$ value for native DNA that is half of value for H-DNA.

**Table 2:** Activation energy parameters resulting from two-state analysis of T-jump kinetics.

Sample	*i*^a^	${\Delta}H_i^\dagger$ (J ∙ mol^−1^)	${\Delta}S_i^\dagger$ (J ∙ mol^−1^ ∙ K^−1^)
DNA	*a*	−2.63 × 10^4^	−1.87 × 10^2^
	*d*	3.77 × 10^5^	9.02 × 10^2^
H-DNA	*a*	−2.48 × 10^5^	−7.54 × 10^2^
	*d*	1.67 × 10^5^	2.46 × 10^2^

The unit is s^−1^ for the dissociation process, and M^−1^ s^−1^ in the DNA association. In the H-DNA association, the units are M^−2^ s^−1^.

^a^*i* = *a* and *d* indicate the association and dissociation process, respectively.

### Effect of ligand binding revealed by the MD simulations

**Fig. 4:**
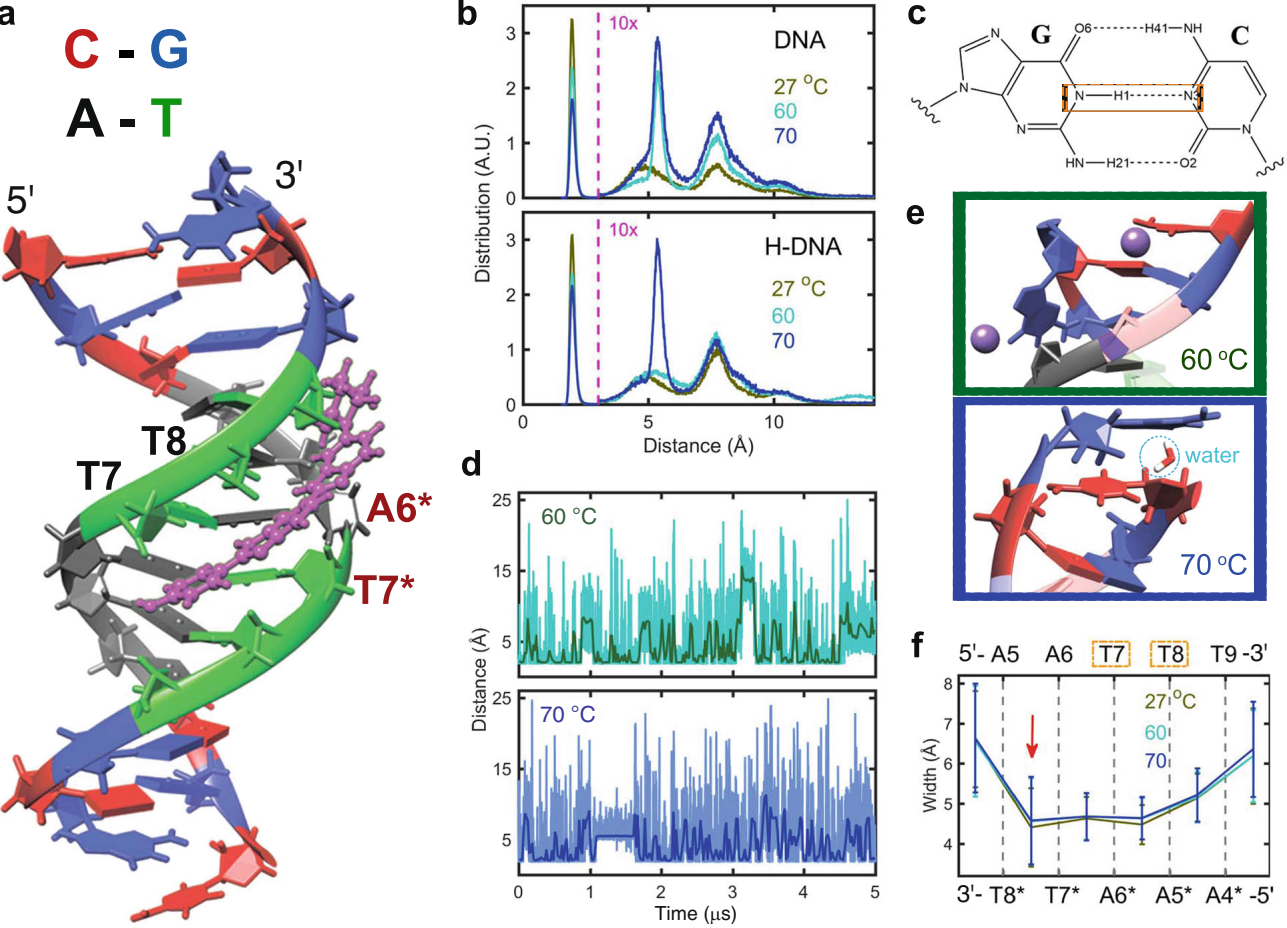
Results of MD simulations. **a** Snapshot of H-DNA complex. Nucleobases C, G, A, and T are color-coded in the DNA duplex. T7, T8, A6*, and T7* are marked as the nucleobases where hydrogen bonds with H are reported by X-ray data^33^. **b** Distribution of the H1–N3 distance in the GC termini shown in (**c**). The distribution with H1–N3 larger than 3 Å (marked with pink dashed lines) are multiplied by a scaling factor of 10. **d** The H1–N3 distance in the GC termini at 60 and 70 °C as a function of the trajectory length. **e** Configuration of the terminal G:C pair when the N1–H3 distance deviates to 15 Å at 60 °C (**e**, upper) and plateaus at ~5.5 Å at 70 °C (**e**, lower). Sodium cations are represented in purple. **f** Average minor-groove widths for the DNA structure throughout the trajectory at 27, 60, and 70 °C. Nucleobases involved in forming hydrogen bonds with H are marked with orange boxes.

For additional insight into the structural and dynamic changes caused by ligand binding, we have employed MD simulations at temperatures of 27, 60, and 70 °C. The minor-groove width, helical propeller, and twist are evaluated according to the 5 µs trajectories to ensure statistical convergence of each system (Supplementary Section 11). The simulations show H preferentially binds to the DNA minor groove through hydrogen bonds involving T7, T8, A6*, and T7*, with its positively charged N-methylpiperazine placing in adjacent to the T9–A4* base pair (Fig. 4a), in agreement with crystallographic studies^33^. In the literature, four hydrogen bonds are generally identified (Supplementary Fig. 22). However, this interpretation has the N–H bonds of H simultaneously donating two hydrogen bonds, which is atypical. In the simulations, we find the hydrogen bonds with T7 and T8 to be dominant, but occasionally hydrogen bonds with T7* and A6* are observed. Essentially, the simulations agree with the standard view that H can form four hydrogen bonds with DNA, but only two satisfy both the distance and angular criterion for hydrogen bonding at any one time (Supplementary Section 13). The binding of H causes an increase of minor-groove width (~1 Å) and uneven width change along the sequence (Supplementary Fig. 18), which has been observed by the NMR and X-ray studies^36,40^. The accommodation of bulky H requires a widened groove for binding, thus causes the distortion of the T8–A6* base-pair step (Supplementary Figs. 19 and 20)^25,41^. With rising temperatures, the minor-groove width at the binding site increases only slightly.

The hydrogen-bond length between the terminal G:C pair provides insight into the fraying event, thus the H1–N3 distance (Fig. 4c) is examined for this purpose. Fraying of the G:C termini is clearly observed with the distribution of H1–N3 distance larger than 3 Å (Fig. 4b). The weakening of duplex stability at the termini with rising temperatures is seen by the fraction of intact hydrogen bonds at this site changing from 23 to 53% between 27 and 70 °C in DNA and 27 to 44% in H-DNA. However, the temperature dependence of H1–N3 distance in H-DNA is less clear, with an unusual distribution at ~14 Å observed at 60 °C and a large amount of H1–N3 distance being ~5.5 Å at 70 °C. In the trajectory for the H1–N3 distance (Fig. 4d), the fraying of G:C termini is seen as spikes with peak values above 3 Å. Each point represents the distance at a single 10 ps snapshot of the trajectory and the solid line displays the averaged value of the snapshots in 5 ns blocks. The large H1–N3 distance of ~14 Å persists for 3–3.2 µs at 60 °C is due to the folding of G base toward the AT tract and stabilized by sodium cations through electrostatic interactions (Fig. 4e). A water molecule plays a role in separating the G:C termini through hydrogen bonding and gives rise to the plateau at ~5.5 Å in the range of 1–1.6 µs at 70 °C.

## Discussion

Surprisingly, the binding of this small ligand only introduces subtle conformational changes to dsDNA but can strongly perturb the duplex dissociation mechanism. Binding of H confers local stability at the binding site but appears to destabilize adjacent bases. At 85 °C (Fig. 1e), all the G:C pairs of H-DNA dissociate within 500 ns while the central A:T pairs remain stable up to milliseconds, emphasizing the prominent interaction between H and the DNA minor groove. The width and width fluctuations in the A6–T9 tract are much smaller upon binding H, indicating that H restricts the dynamic movement of the DNA minor groove^25^. This is caused by the hydrogen bonds of H with the T7 and T8 carbonyls and electrostatic interactions between the DNA backbone and the charged N-methylpiperazine adjacent to the T9–A4* base pair. Compared to the T8–T9 tract, the deviation of width in the A5–A6 tract is ~60% larger and increases by 10% instead of 3% by raising the temperature to 60 °C (Fig. 4f).

Although the fraying of the terminal G:C base pairs has been previously observed in both native and H-DNA^25,30^, the effect of ligand binding on this process is elusive. Recently, Fritzsch et al.^31^ reported a dissociation kinetics study of H bound to similar duplexes that vary in their central AT sequences using T-jump IR spectroscopy, finding that the ligand’s impact on fraying is correlated with its binding affinity. As with our results, they observed that H binding suppressed the short time dissociation response (~100 ns) from A ring modes. However, in contrast to our results, they concluded that end-fraying is suppressed by ligand binding based on the observation that the ~100 ns fraying of the G:C termini showed a larger change in DNA without H bound than for H-DNA. We believe these conclusions are influenced by the observation window for the experiment, which was limited to ~100 μs, when the full dissociation time-scales we observe are more than an order of magnitude slower.

As discussed previously, the peak frequency of the T carbonyls shifts from 1698 to 1702 cm^−1^ upon hydrogen-bond formation with H. This binding signal is located in a frequency range away from the dissociation features, making it a good indicator for monitoring these hydrogen bonds. The spectral change of the H–T breaking appears before the average duplex dissociation time in the transient 2D-IR spectra, and its observed rate is consistently larger than the dissociation response of the A:T pairs (Supplementary Fig. 12). These observations suggest that water molecules interrupt the hydrophobic region in H-DNA by breaking the hydrogen bonds of H with the T carbonyls. The rate distribution of this binding signal and the A:T dissociation response highlights the significance of the hydrophobic interactions in stabilizing the complex, rather than the hydrogen bonds, although the electrostatic attraction between the charged ligand and DNA backbone is also an important stabilizer of the minor-groove structure in the T8–T9 tract.

Our equilibrium results show that the association of H-DNA from its three constituents is well described by a two-state model. In contrast, by employing a three-state model we find that the calculated binding constant of H with D at 25 °C is highly dependent on *C*_D_, with its value increasing by two orders of magnitude to 1.4 × 10^7^ M^−1^ upon reducing *C*_D_ from 1 mM to 200 nM (Supplementary Table S3). Furthermore, the [DH] calculated using the three-state model does not scale linearly with the fluorescence intensity of H-DNA solutions as the concetration is changed (Supplementary Fig. 7); however, for the two-state model it does. Especially under the condition that the fraction of ssDNA is >1% in the absence of H, the calculated [DH] using two-state is obviously larger than the value suggested by three-state model. Therefore, we conclude that the association of H-DNA is a concerted binding process that is facilitated by the electrostatic interactions between H and nearby ssDNA (Supplementary Fig. 15). By tuning the screening effect with increased ionic strength in the buffer, the overall binding affinity shows a clear decrease, suggesting that electrostatic contributions are important in the formation of H-DNA^12^. The diffusion coefficient of H (8.3 × 10^−5^ cm^2^ s^−1^)^42^ is 68 times larger than that of the dsDNA used in this study (1.22 × 10^−6^ cm^2^ s^−1^) at room temperature^43^, indicating that H is likely to sample a variety of nonspecific binding configurations with single strands as they approach each other during association. Free-flow electrophoresis studies show that the diffusion coefficient of both ssDNA and dsDNA follows Zimm’s theory that is determined by the molecular size^44^, meaning the diffusion coefficient of ssDNA is only somewhat larger than 1.22 × 10^−6^ cm^2^ s^−1^. The prominent mobility of H helps in forming and stabilizing the critical nucleus, which is essential in the hybridization of canonical DNA. Conformational changes of DNA and displacement of spine of hydration, are considered as the last step in forming a stable H-DNA complex that are sequence-dependent due to the superior accommodation of H^26^.

In summary, we have demonstrated that the small ligands binding can strongly disturb the stability of base pairs adjacent to the binding site, with the observation that the percentage of dissociated G:C pairs is 66% in H-DNA instead of 17% in native DNA within the first 100 ns after T-jump. Such observation is very striking, given the strong stabilization effect of ligand on dsDNA which increases the melting temperature by 45 °C and dramatically slows down the dissociation of dsDNA with the observed time being 50 times larger than that of native DNA. The hydrogen bonding is found to be essential in stabilizing H-DNA, by seeing that the hydrogen bonds between H and thymines break prior to the dissociation of central A:T pairs. Our thermodynamic analysis suggests that H works concertedly with ssDNA in the formation of H-DNA, rather than binding directly to dsDNA. Under conditions that dsDNA is slightly unstable (the fraction of ssDNA >1%), the H+dsDNA association description shows obvious failure in predicting the fraction of H-DNA, indicating that the association of H-DNA is a concerted process. The association/dissociation free energy change is more appropriate than the binding constant, for characterizing the binding affinity between ligands and the DNA binding site. The experimental and analytical approach developed here can further help in elucidating the binding mechanism of other DNA binders, and provide a different perspective in the design of new type derivatives for extensive applications in biotechnology.

## Methods

### Sample preparation

The DNA oligonucleotide used in this study is the self-complementary sequence 5′-CGC AAA TTT GCG-3′, purchased from Integrated DNA Technologies (IDT) at desalt-grade purity and further purified by dialysis using membrane tubing with a 0.5 kD MWCO (Spectrum Micro Float-A-Lyzer) and distilled water at 0 °C for 8 h. Hoechst 33258 (H) was purchased from Chemodex and is used as received. The duplex DNA bound with Hoechst 33258 is denoted by H-DNA. To avoid the formation of DNA hairpins, all DNA samples were prepared in pH 7.4 deuterated buffer with 5 mM sodium phosphate and 200 mM sodium chloride. pH values were adjusted with 1 M HCl and NaOH solutions and then checked with pH meter (Fisherbrand accumet AB150). The concentration of oligonucleotide and H was confirmed on a NanoDrop UV/vis spectrometer (Thermo Scientific). H-DNA complexes were prepared by mixing buffer solutions in a 1:1 dsDNA-to-H mole ratio. We use *C*_D_ to refer to the total concentration of native and H-DNA (the equivalent value of dsDNA). Prior to measurements, all samples were annealed by heating to 95 °C for 3 min and then cooling gradually to room temperature over 10–15 min. For IR measurements, labile protons of DNA and H were HD exchanged in deuterated water (D_2_O, Cambridge Isotopes) and lyophilized before dissolving into the deuterated buffer.

### Fluorescence spectroscopy

Fluorescence experiments were carried out using a Horiba Fluorolog-3 at 1 nm resolution. The samples were excited at 345 nm. The temperature-dependent fluorescence was recorded at 446 nm. The 0.2 and 2 µM samples were prepared via serial dilution from the original 1 mM solution. A quartz cuvette with 0.5 cm path length is used for all fluorescence measurements.

### Temperature-ramp FTIR spectra

For all IR spectroscopy, samples were held between two CaF_2_ windows with a 50 μm Teflon spacer defining the path length and mounted in a home-built brass sample cell connected to a recirculating chiller to control the sample temperature. In the FTIR measurements, the bath temperature was ramped between 0 and 105 °C in 3 °C steps, and the temperature of the sample was recorded with a thermocouple embedded in the brass sample jacket, resulting in a range of 5–100 °C. Spectra were recorded on a Bruker Tensor 27 FTIR spectrometer at 4 cm^–1^ resolution, averaging 30 scans at each temperature step after waiting 75 s for the sample cell to equilibrate at each bath set point.

### Nonlinear IR spectroscopy

The instrumentation and methods for acquiring steady-state and transient nonlinear IR data have been described previously^37^. Briefly, experiments are performed in the boxcar geometry with three variably time-delayed pulses generating the nonlinear signal and a fourth reference pulse used for balanced heterodyne spectral interferometry. The nonlinear signal was collected at a fixed waiting time *τ*_2_ = 150 fs as a function of evolution time *τ*_1_ scanned in 4 fs steps out to 2500 and 2000 fs for rephrasing and non-rephasing spectra, respectively. The transient heterodyned dispersed vibrational echo (t-HDVE) and transient 2D-IR (t-2D-IR) data were collected over a time window from 5 ns to 50 ms after a 15 °C T-jump. To extend the observation time window, which is limited by the thermal re-equilibration time of solvent, we coated the CaF_2_ windows with 12.5 µm Dupont FEP fluorocarbon film using vacuum oven (Fisherbrand Isotemp Model 281A). An optical quality coating was achieved by heating the film attached windows at 290 °C for 30 min under vacuum. The t-HDVE spectra were collected using the Fourier transform spectral interferometry method, with the local oscillator stepped from −10 to 10 fs in 5 fs steps. For t-2D-IR data collection, *τ*_1_ was undersampled in 16 fs steps from −60 fs to 1750 and 1250 fs for rephasing and non-rephasing surfaces, respectively. t-HDVE time traces and t-2D-IR surfaces represent at least five averaged data sets. The polarization was set to ZZZZ for all nonlinear IR measurements.

### Molecular dynamics simulations

All simulations are performed using the Amber 18.0^45^ package with the bsc1 force field^46^ applied to the double stranded DNA sequence 5′-CGC AAA TTT GCG-3′. The SPC/E water model^47^ is utilized and H has been previously parameterized by Furse et al.^48^. Following the extensive equilibration protocol described in Supplementary Materials and Methods Section, production simulations are run for 5 µs under constant volume and constant temperature conditions and snapshots are collected every 10 ps for analysis with Cpptraj^49^ and Curves+^50^.

### Statistics and reproducibility

All anlaysis and simulations were performed using available softwares and packages mentioned in the “Methods”. For the fluorescence measurements, sample size 1 mL was used and each data point is the average of five replicates. For the infrared measurements, sample size was 27 µL. The number of replicates is 32 for the equilibrium IR spectra and 8 for the transient IR spectra. All the above mentioned experimental dataset were repeated at least once to confirm their reproducibility.

### Reporting summary

Further information on research design is available in the Nature Research Reporting Summary linked to this article.

## Supplementary information

Supplementary Information

Description of additional supplementary files

Supplementary Data 1

Reporting Summary

## Data Availability

All data supporting the findings of this study are available within the Article and its Supplementary Information and/or from the corresponding author upon reasonable request. Source data underlying plots shown in figures are provided in Supplementary Data 1.
