# Supplementary material for: DNA minor-groove binder Hoechst 33258 destabilizes base-pairing adjacent to its binding site
Source: Commun Biol. 2020 Sep 22;3:525. doi: 10.1038/s42003-020-01241-4 (PMC7508854; doi:10.1038/s42003-020-01241-4)
Supplement: Supplementary file 1 — Supplementary Information [file 42003_2020_1241_MOESM1_ESM.pdf]

Supplementary Figures

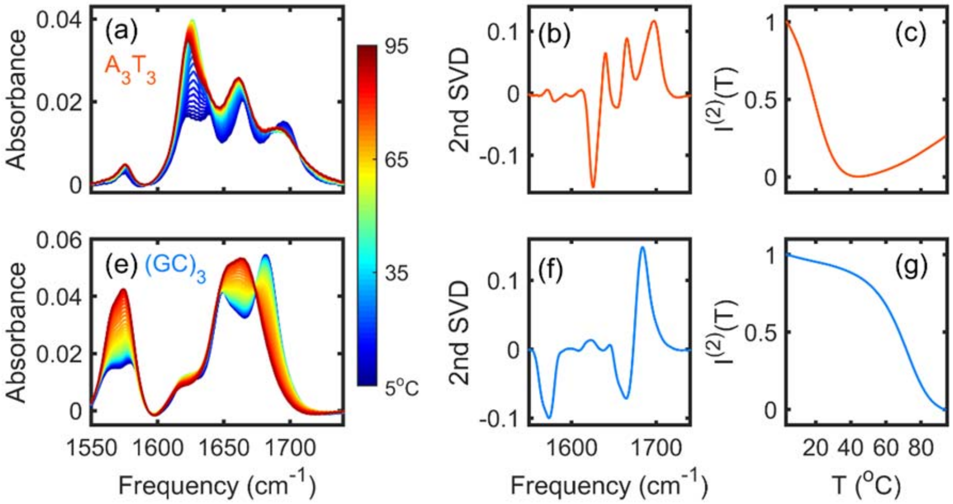

Supplementary Fig. 1 Temperature ramp FTIR spectra from 5 to 95 °C with 2 mM A_3T_3 and $(GC)_3$duplex. (a) A_3T_3 in 10 mM Tris, 200 mM NaCl and 50 mM $MgCl_2$, pH 7.4 solution, and its second SVD (b) spectral and (c) amplitude component. (e) $(CG)_3$ in 10 mM Tris, 200 mM NaCl, pH 7.4 solution, and its second SVD (f) spectral and (e) amplitude component.

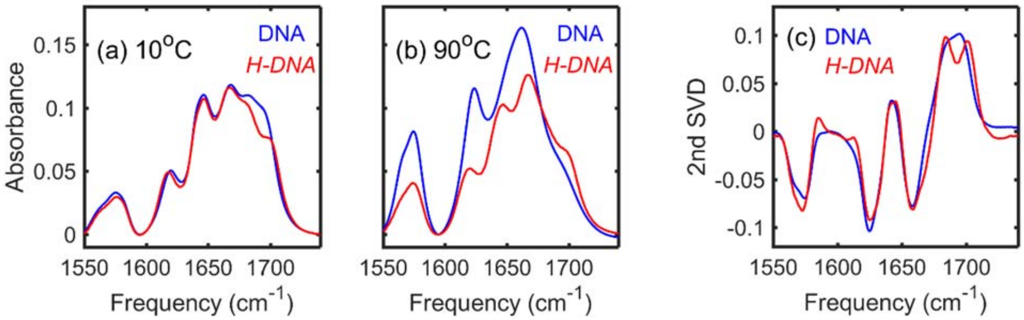

Supplementary Fig. 2 FTIR spectra of 1 mM DNA and *H-DNA* complex in 5 mM Na_2DPO_4, 200 mM NaCl, pH 7.4 buffer measured at (a) 10 °C and (b) 90 °C. (c) second SVD spectral component of temperature ramp spectra in the range of 5-95 °C.

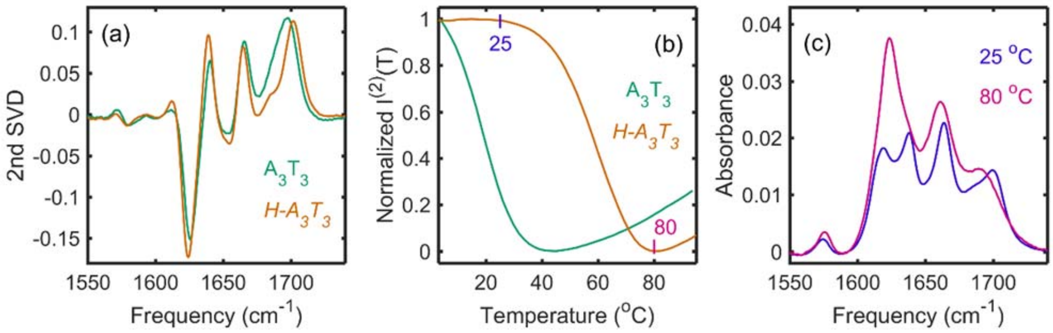

Supplementary Fig. 3 Second SVD components of temperature ramp FTIR spectra of 2 mM A_3T_3 and H-A_3T_3 complex in 10 mM Tris, 200 mM NaCl and 50 mM $MgCl_2$, pH 7.4 solution (a) spectral and (b) amplitude component. (c) spectra of H-A_3T_3 complex measured at 25 and 80 °C.

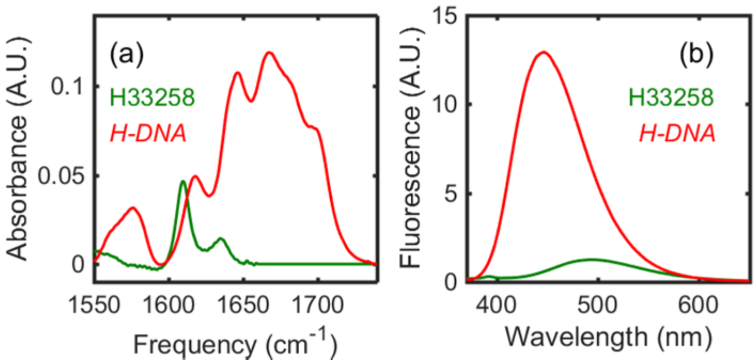

Supplementary Fig. 4 IR (a) and fluorescence (b) spectra of *H* and *H-DNA* at 25 °C. The concentration ratio between *H* and *H-DNA* is 30:1.

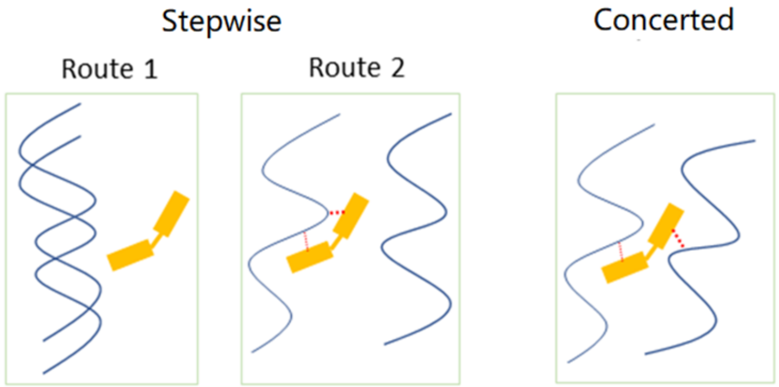

Supplementary Fig. 5 Binding scenarios of *H-DNA* complex.

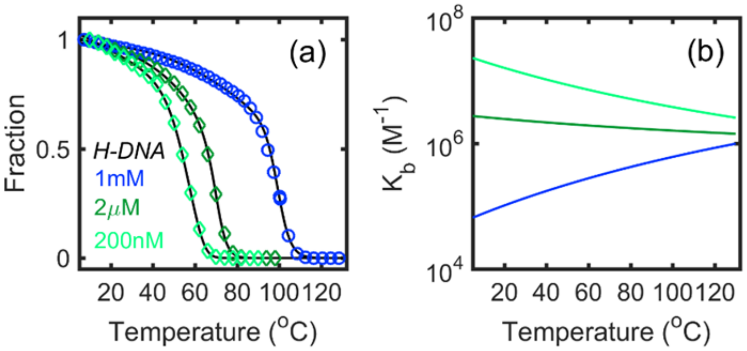

Supplementary Fig. 6 Three-state analysis of the melting curves of *H-DNA*. (a) Melting curve obtained with SVD analysis of the FTIR spectra for 1mM *H-DNA* (circles) and normalized temperature-dependent fluorescence intensity at 446 nm for 200nm and 2µM *H-DNA* (diamonds). The fitted curves obtained from a three-state model described by Eq. S7 and S8, are shown with black solid lines. (b) The binding constants obtained by fitting the color-coded data points in (a) using Eq. S9.

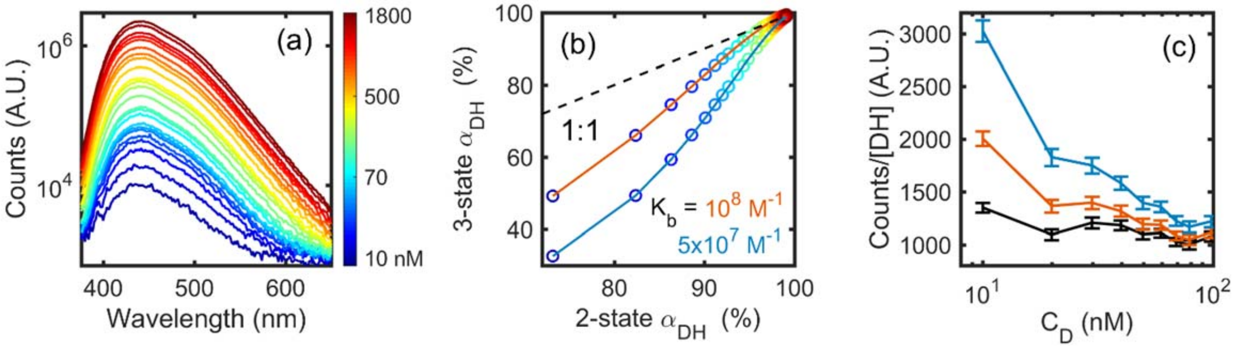

Supplementary Fig. 7 Fluorescence titration measurement performed at 40 °C with C_D ranging from 10 nM to 1.82 µM. (a) Fluorescence spectra of *H-DNA* solution with various C_D. (b) Fraction of *DH* calculated by using 2-state (Eq.S14) and 3-state model (Eq. S7 and S8) with K_b equals to 10^8 (orange) and $5×10^7$ M^{-1} (blue). Data points are color-coded as spectra in (a). Dashed line shows the reference when the two calculated fractions are equal. (c) Correlation of C_D with the averaged peak counts (427-454 nm) per mole *DH*. Lines associated with [*DH*] calculated using 3-state model with K_b equals to 10^8 (orange) and $5×10^7$ M^{-1} (blue). The black line represents the correlation predicted by 2-state model. Error bars represent the standard deviation of the averaged counts divided by [DH].

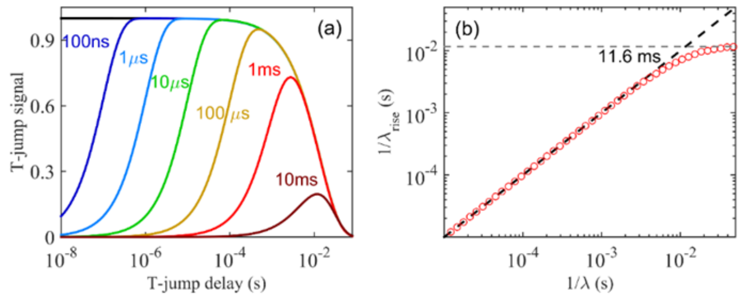

Supplementary Fig. 8 Deconvoluted relaxation times obtained from input rise times. The observed signal resulting from convolution (a) is fit to a bi-exponential with a rise time of $1/\lambda_{rise}$. The corresponding deconvoluted relaxation time, $1/\lambda$, is obtained from the 1 μs correlation line (b).

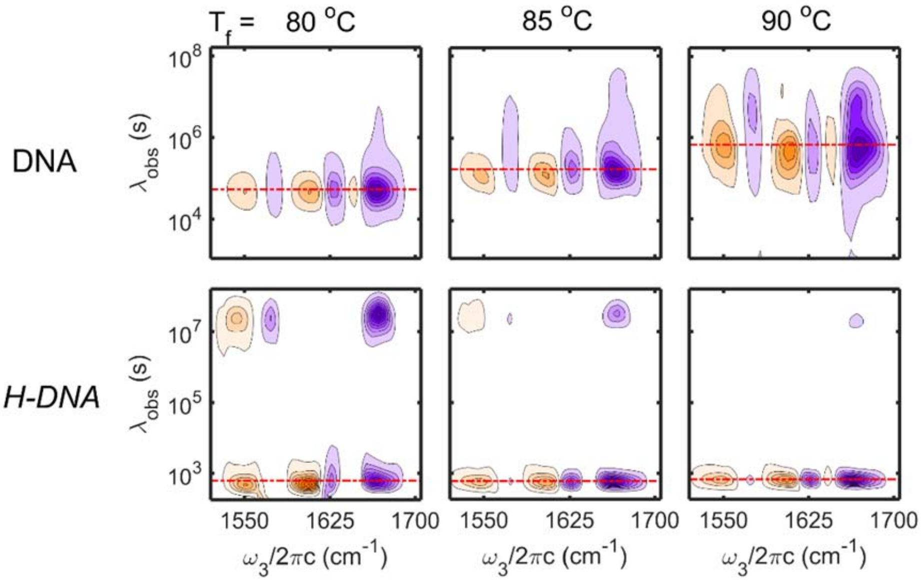

Supplementary Fig. 9 MEM rate distributions of T-jump data of DNA and *H-DNA* measured at final temperature 80, 85 and 90 °C, with ΔT = 15 °C. Orange and purple contours represent positive and negative rate amplitude, respectively. Dashed lines indicate the position of average observed rate λ_{obs} of dissociation process.

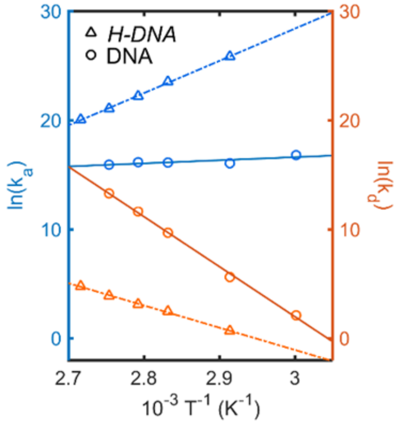

Supplementary Fig. 10 Arrhenius plots derived from two-state analysis of the dissociation process of *H-DNA* (triangles) and DNA (circles).

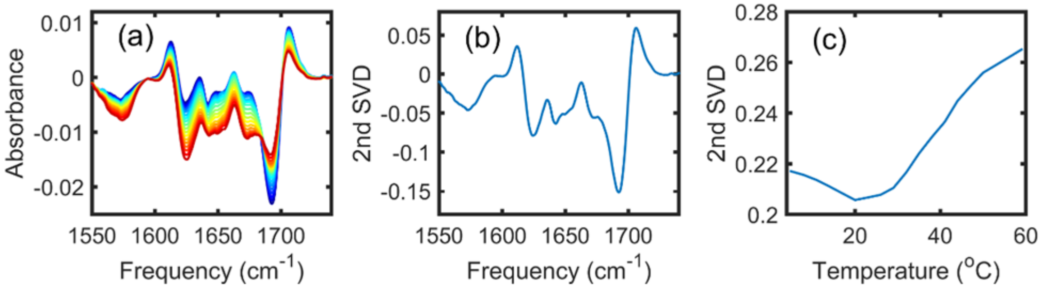

Supplementary Fig. 11 (a) The difference between the FTIR spectra of 1 mM *H-DNA* with 1 mM DNA in 5 mM Na_2DPO_4, 200 mM NaCl, pH 7.4 buffer measured over the 5-60 ^oC temperature range, where DNA is primarily in its duplex form. The loss of signal at 1698 cm^{-1} and gain at 1702 cm^{-1} reflect the blue shift of the $T(C^2=O)$ carbonyl upon binding *H*. The second SVD spectral component and the amplitude of the difference spectra in (a) are more clearly illustrated in (b) and (c), respectively.

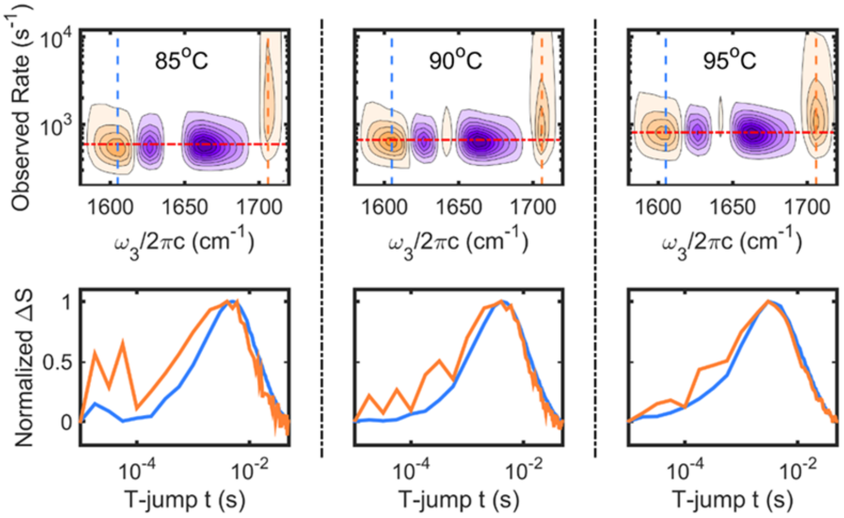

Supplementary Fig. 12 (Top) MEM rate distributions of t-HDVE data of *H-DNA* measured at final temperature 85, 90, and 95 °C with T = 15 °C. Orange and purple contours represent positive and negative rate amplitude, respectively. Orange dashed lines indicate the position of averaged observed rate of the dissociation process. The rate amplitude at frequencies above 1700 cm^{-1} is scaled by a factor of 15. (Bottom) Normalized t-HDVE time traces at 1607 and 1706 cm^{-1}, indicated with color-coded dashed lines in the upper panel.

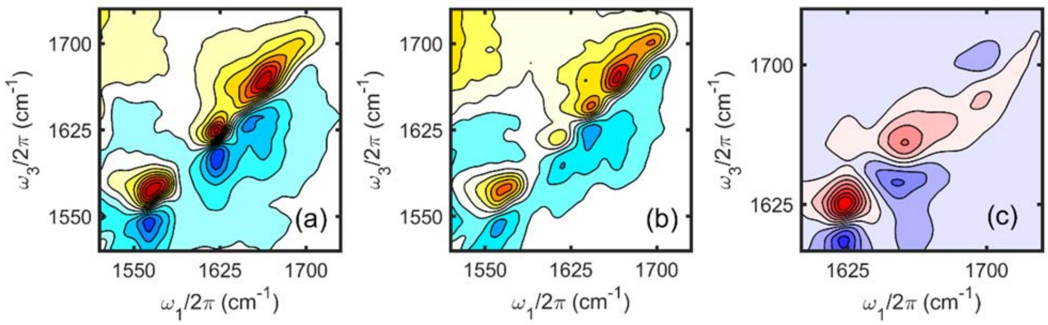

Supplementary Fig. 13 2D IR surfaces of 1 mM (a) DNA, (b) *H-DNA*, and their difference spectrum (c) measured at 70 °C with parallel polarization.

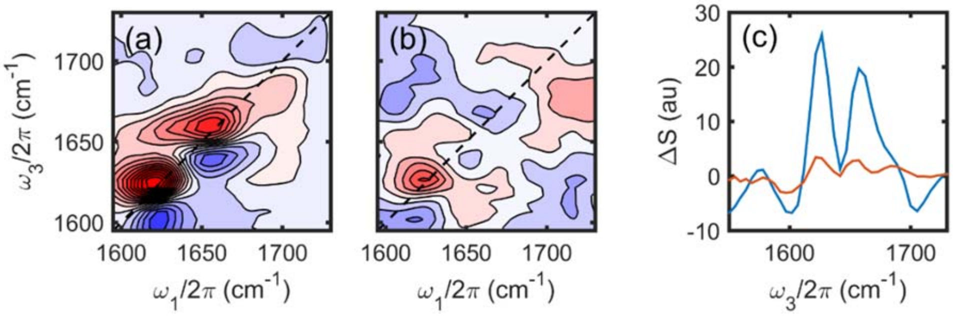

Supplementary Fig. 14 Transient 2D IR difference spectra of 1 mM *H-DNA* (a) 2 ms – 500 ns, (b) 4 ms – 2 ms, and their (c) projections onto the ω_3 axis of difference t-2D IR surface shown in (a) blue and (b) red.

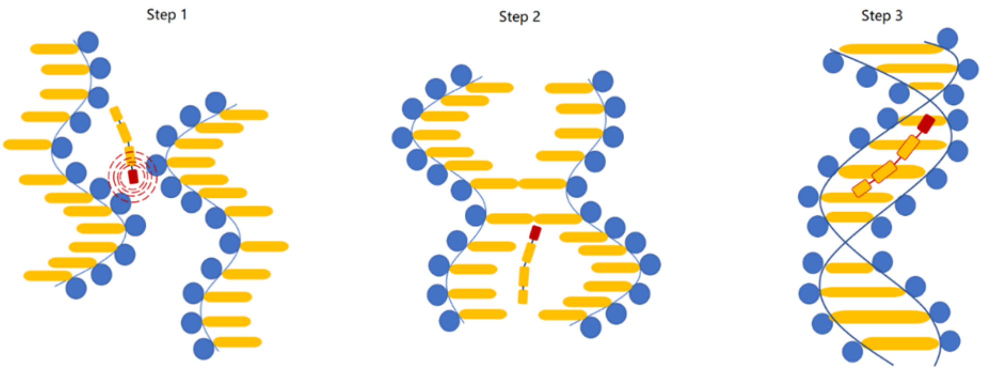

Supplementary Fig. 15 Schematic diagram of the binding mechanism between *H* and ssDNA. The red square represents the positively charged end of *H*.

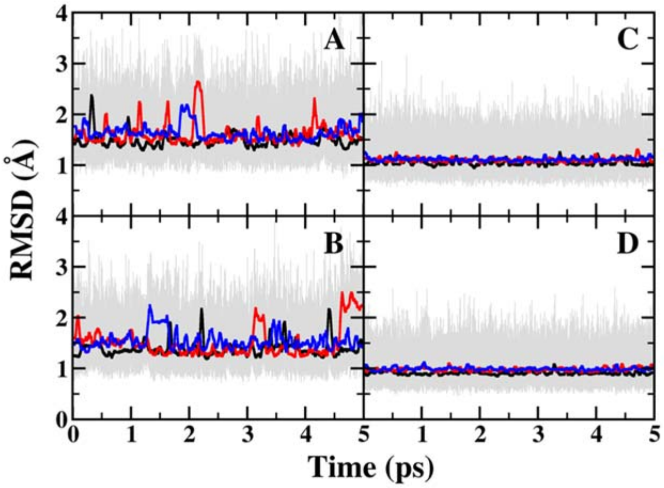

Supplementary Fig. 16 The calculated root mean-squared displacements (RMSDs) for the DNA and *H-DNA* strands at 300 K (black), 333 K (red), and 343 K (blue) with respect to the average structure of the stand over the 5 μs trajectory. The RMSD for all 12 base pairs of DNA (A) and *H-DNA* (B) show the expected jumps associated with fraying at the ends of the strand. When the RMSDs are evaluated for the internal 8 base pairs, DNA (C) and *H-DNA* (D), no jumps are present.

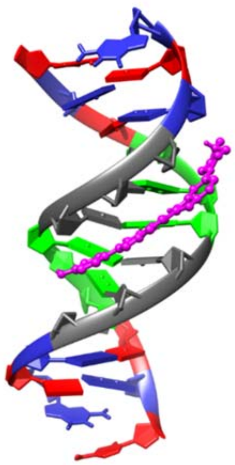

Supplementary Fig. 17 An illustration of *H* (pink) bound to the DNA strand, where the bases are color coded: C (red), G (blue), A (green), and T (grey).

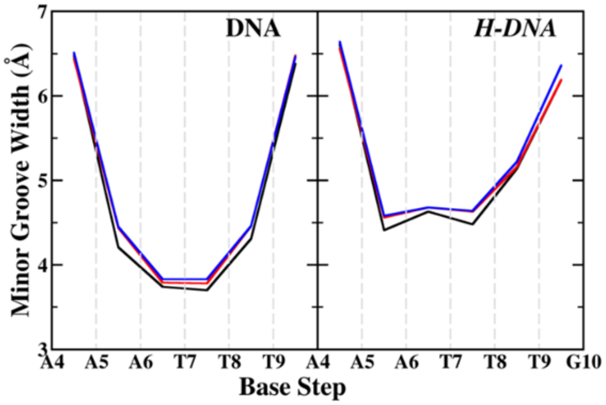

Supplementary Fig. 18 The calculated minor groove width for the DNA (left) and *H-DNA* (right) at 300 K (black), 333 K (red), and 343 K (blue).

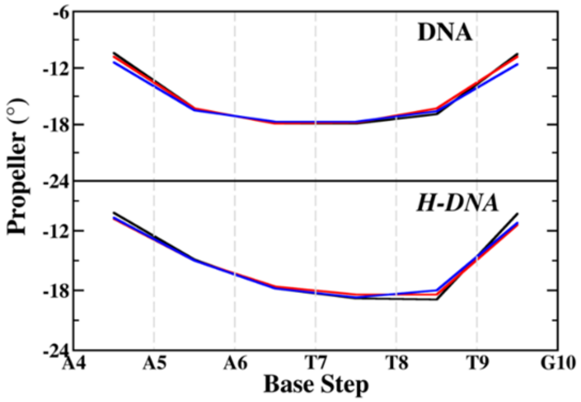

Supplementary Fig. 19 The calculated helical propeller for DNA and *H-DNA* at 300 K (black), 333 K (red), and 343 K (blue).

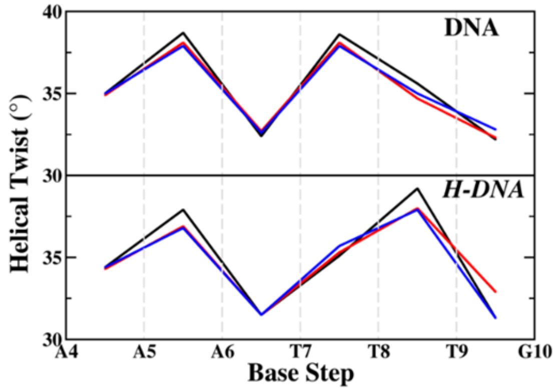

Supplementary Fig. 20 The calculated helical twist for DNA and *H-DNA* at 300 K (black), 333 K (red), and 343 K (blue).

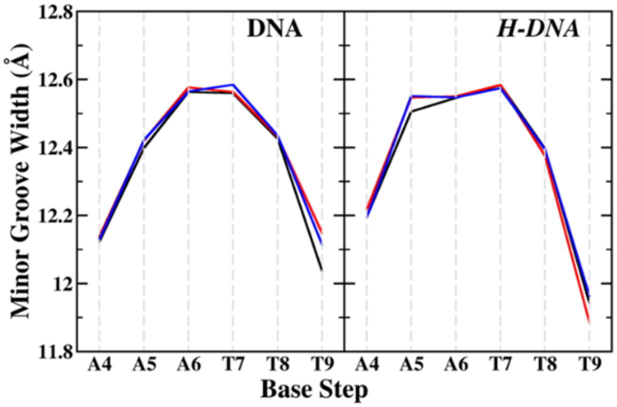

Supplementary Fig. 21. The calculated minor groove width using the O4 – O4 distance as the metric for the DNA (left) and *H-DNA* (right) at 300 K (black), 333 K (red) and 343 K (blue).

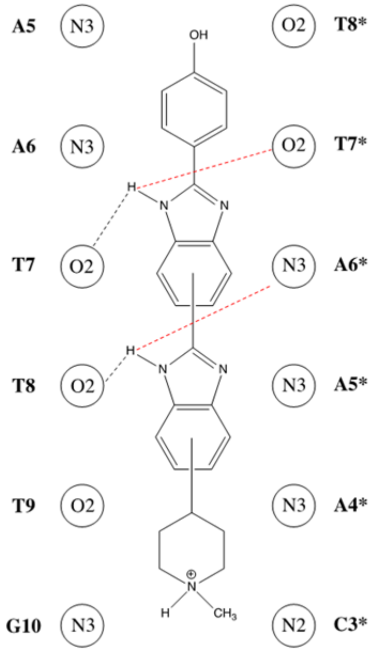

Supplementary Fig. 22 Schematic representation of hydrogen-bond interactions in *H-DNA*.

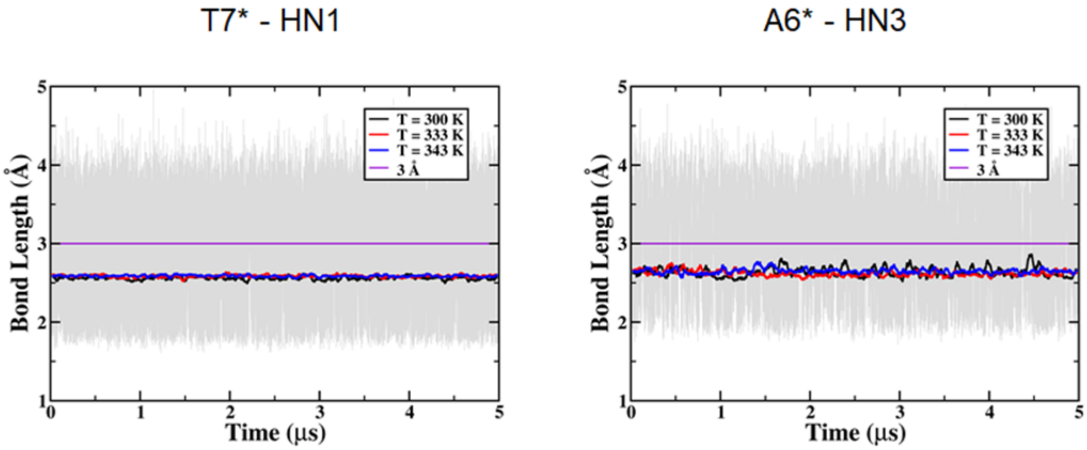

Supplementary Fig. 23 The calculated hydrogen-bond length of *H* with T7* (left) and A6* (right). The cutoff length (3 Å) is shown with purple lines.

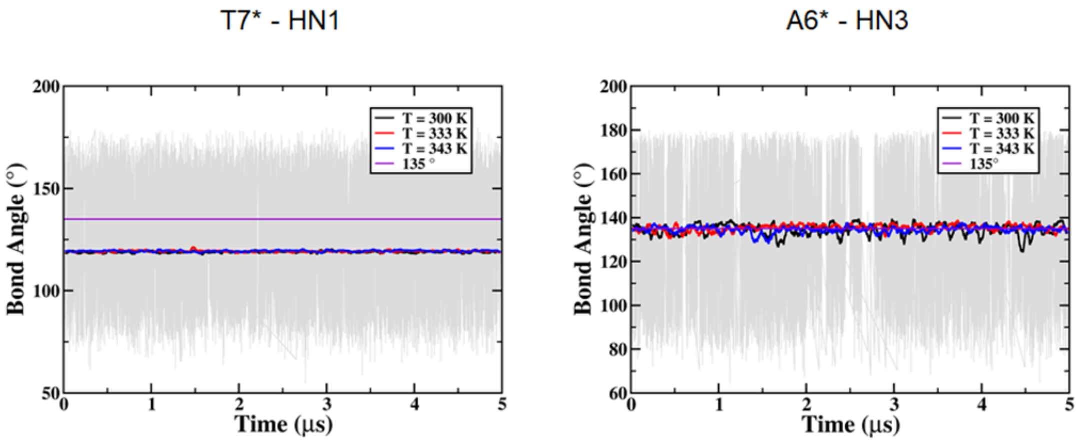

Supplementary Fig. 24 The calculated hydrogen-bond angle of *H* with T7* (left) and A6* (right). The cutoff angle (135 ^{o}) is shown with purple lines.

Supplementary Tables

Supplementary Table 1.ystem Details of the MD Simulations

System	Box Volume ($Å^3$)	# of Na^+ Ions	# of Cl^- Ions	# of H_2O
DNA	255822	49	27	5433
H-DNA	225975	48	27	5429

Supplementary Table 2. Peak Frequencies of the Spectral Features Correlated with Duplex Formation

Sequences	Positive Signal (cm^{-1})	Negative Signal (cm^{-1})
A_3T_3	1640, 1666, 1698	1627
$(GC)_3$	1684	1574, 1665

Supplementary Table 3. Thermodynamics Parameters for Melting Transition

Sample	C_D (M)	ΔH_b^0 (kJ mol^{-1})	ΔS_b^0 (J mol^{-1} K^{-1})	K_b (M^{-1}) at 25 °C
H-DNA	1×10^{-3}	20.2	165	1.2×10^{5}
	2×10^{-6}	-4.8	106	2.4×10^{6}
	2×10^{-7}	-16.3	82	1.4×10^{7}

Supplementary Table 4. Observed Rates and Two-State Rate Constants

Sample	T_f (^oC)	λ_{obs} (s^{-1})*	θ_{int}(%)	θ_{ext}(%)	K_d (M)	k_a ($M^{-1}s^{-1}$)	k_d (s^{-1})
DNA	60	1.6×10^{3} (±8%)	77	99	4.3×10^{-7}	1.93×10^{7}	8.35×10^{0}
	70	6.5×10^{3} (±2%)	73	92	3.0×10^{-5}	9.35×10^{6}	2.81×10^{2}
	80	5.3×10^{4} (±2%)	69	53	1.6×10^{-3}	9.87×10^{6}	1.62×10^{4}
	85	1.8×10^{5} (±3%)	67	22	1.1×10^{-2}	1.02×10^{7}	1.14×10^{5}
	90	6.5×10^{5} (±11%)	65	5	7.2×10^{-2}	8.21×10^{6}	5.92×10^{5}
					K_d (M^2)	k_a ($M^{-2}s^{-1}$)	k_d (s^{-1})
H-DNA	70	4.1×10^{2} (±6%)	79	100	1.2×10^{-11}	1.64×10^{11}	1.94×10^{0}
	80	6.2×10^{2} (±4%)	75	94	7.3×10^{-10}	1.63×10^{10}	1.19×10^{1}
	85	6.0×10^{2} (±3%)	73	89	5.2×10^{-9}	4.30×10^{9}	2.25×10^{1}
	90	6.6×10^{2} (±2%)	71	81	3.6×10^{-8}	1.38×10^{9}	4.92×10^{1}
	95	8.1×10^{2} (±2%)	69	66	2.3×10^{-7}	5.10×10^{8}	1.18×10^{2}

* Deviation of average λ_{obs} is the amplitude-weighted standard deviation of λ_{obs} across the entirety of the dissociation response.

Supplementary Table 5. Activation Energy Parameters Resulting from Arrhenius Analysis

Sample	A_a(*)	E_a (kJ mol^{-1})	A_d (s^{-1})	E_d (kJ mol^{-1})
DNA	3.2×10^{3}	-24	3.2×10^{60}	381
H-DNA	7.1×10^{-27}	-246	1.2×10^{26}	169

* units are $M^{-1}s^{-1}$ for DNA, while $M^{-2}s^{-1}$ for *H-DNA*.

Supplementary Table 6. The calculated minor groove widths and standard deviations for each base step at T = 300 K for DNA and *H-DNA*

	DNA		*H-DNA*	
Base Step	Minor Groove Width	Standard Deviation	Minor Groove Width	Standard Deviation
4A – 5A	6.48 Å	1.34 Å	6.61 Å	1.20 Å
5A – 6A	4.21 Å	1.35 Å	4.41 Å	0.98 Å

6A – 7T	3.74 Å	1.06 Å	4.63 Å	0.54 Å
7T – 8T	3.70 Å	1.06 Å	4.48 Å	0.49 Å
8T – 9T	4.31 Å	1.26 Å	5.14 Å	0.61 Å
9T – G10	6.38 Å	1.41 Å	6.19 Å	1.19 Å

Supplementary Table 7. The calculated minor groove widths and standard deviations for each base step when T = 333 K for DNA and *H-DNA*

	DNA		*H-DNA*	
Base Step	Minor Groove Width	Standard Deviation	Minor Groove Width	Standard Deviation
4A – 5A	6.44 Å	1.50 Å	6.56 Å	1.38 Å
5A – 6A	4.44 Å	1.40 Å	4.56 Å	1.08 Å
6A – 7T	3.79 Å	1.12 Å	4.68 Å	0.58 Å
7T – 8T	3.78 Å	1.13 Å	4.63 Å	0.52 Å
8T – 9T	4.46 Å	1.39 Å	5.16 Å	0.63 Å
9T – 10G	6.48 Å	1.48 Å	6.19 Å	1.15 Å

Supplementary Table 8.The calculated minor groove widths values and standard deviations for each base step when T = 343 K for DNA and *H-DNA*

	DNA		*H-DNA*	
Base Step	Minor Groove Width	Standard Deviation	Minor Groove Width	Standard Deviation
4A – 5A	6.51 Å	1.46 Å	6.64 Å	1.36 Å
5A – 6A	4.45 Å	1.42 Å	4.58 Å	1.09 Å
6A – 7T	3.83 Å	1.18 Å	4.68 Å	0.59 Å
7T – 8T	3.83 Å	1.16 Å	4.64 Å	0.53 Å
8T – 9T	4.46 Å	1.39 Å	5.22 Å	0.66 Å
9T – 10G	6.46 Å	1.47 Å	6.36 Å	1.19 Å

Supplementary Notes

1. Overview of the AT and GC Base Pair Melting

The temperature dependent FTIR spectra of A_3T_3 and $(GC)_3$DNA sequences are shown in Supplementary Fig. 1. At low temperature, the spectra of both DNA sequences have five dominant peaks that are frequency shifted and broadened by the increasing temperature due to the dissociation of CG or AT base pairs. The singular value decomposition (SVD) of the FTIR spectra provides insights into the temperature-dependent changes of the structure with the amplitude components shown in Supplementary Fig. 1c and its corresponding spectral features in Supplementary Fig. 1b. Compared to $(GC)_3$, the A_3T_3 DNA sequence is very unstable. Even in the presence of 50 mM Mg^{2+}, the inflection point of its SVD amplitude component is ~55 ^{o}C lower than that of $(GC)_3$ sequence. Supplementary Table 2 summarizes the observed spectral features correlated with the dissociation of the A_3T_3 and $(GC)_3$ sequences. As illustrated in Supplementary Fig. 1b and f, upon the duplex formation, the most dramatic intensity loss of A_3T_3 appears at 1627 cm^{-1}, which is assigned to the A ring mode. In contrast, the two negative spectral changes of $(GC)_3$ observed at 1574 and 1665 cm^{-1} are due to the G ring mode and carbonyl mode, respectively.

2. Spectral Characterization of Ligand Binding and its Effect on DNA Stability

The temperature-dependent FTIR spectra of DNA and *H-DNA* in pH 7.4 solution are shown in Supplementary Fig. 2. At 10 ^{o}C, both DNA and *H-DNA* are predominantly dimers, thus the difference between the two spectra in Supplementary Fig. 2a is primarily caused by the induced structural change or loss of hydration by ligand binding.[11] At the binding interface, three thymine (T) carbonyls pointing to the minor groove, are hydrogen bonded to *H* instead of the water molecules in native DNA structure, giving rise to the dramatic change in its vibrational mode. A clear shoulder peak appears at ~1702 cm^{-1}, which originates from the T carbonyl stretching mode at ~1698 cm^{-1}. The intensity drop of this mode suggests the interaction between *H* and DNA is weaker than its hydrogen bonding with water;[11] however, the *H* and DNA interaction is still sufficient to preserve the duplex at high temperature. As shown in Supplementary Fig. 2b, with an increased temperature to 90 ^{o}C, the spectral intensity of *H-DNA*

at 1574 and 1627 cm^{-1} varies slightly, while native DNA is nearly doubled indicating a monomer dominant state. The melting spectral features of native and *H-DNA*, are similar with small differences at ~1695 cm^{-1} due to the T carbonyl mode.

For a clearer characterization of the effect of ligand binding to the T carbonyl mode, we have measured the A_3T_3 and *H-*A_3T_3 complex. The second SVD component in Supplementary Fig. 3a, representing the spectral features upon melting, shows a clear frequency shift from 1698 to 1702 cm^{-1} in the presence of *H*. The inflection point of melting transition curves in Supplementary Fig. 3b, increases from ~20 ^{o}C to ~60 ^{o}C due to the stabilized duplex structure by ligand. With the temperature rising to 80 ^{o}C, as illustrated in Supplementary Fig. 3c, the shoulder peak at 1701 cm^{-1} shifts back to 1691 cm^{-1}, resulting from the breaking of the hydrogen-bond between *H* and T carbonyls and weakening of the hydrogen-bond network at high temperatures.

3. IR and Fluorescence Spectra of H33258 and *H-DNA*

As shown in Supplementary Fig. 4a, the IR spectrum of *H* contains two dominant peaks at 1610 and 1635 cm^{-1}. Compared to the absorption of *H-DNA*, the intensity of *H* at these two frequencies is ~20 times weaker, thus is barely detectable with the same concentration of DNA.

The fluorescence spectrum of free ligand *H* is peaked at 493 nm. Upon binding to the DNA minor groove, as illustrated in Supplementary Fig. 4b, the peak wavelength shifts to 446 nm with the intensity increases by ~100 times. Therefore, the association/dissociation process of *H-DNA* can be monitored by tracking the fluorescence intensity change.

4. Discussion on the Binding Scenario of *H-DNA*

The melting curves $\theta(T)$ obtained with T-ramp FTIR or fluorescence spectra provide fraying and dissociation as independent processes, which can be described by the internal (θ_{int}) and external (θ_{ext}) fractions of intact base pairs present,[12]

$$\theta(T) = \theta_{\text{int}}(T)\,\theta_{ext}(T) \qquad \text{(S1)}$$

$$\theta_{int} = \frac{[c]}{L[D]} = \frac{1}{1+K_f} \quad \text{(S2)}$$

$$\theta_{ext} = \theta_D = \frac{[D]}{C_D} \quad \text{(S3)}$$

Here, [c] is the concentration of base pairs and L is the length of DNA duplex. We use C_D to refer to the total concentration of native and *H-DNA* (the equivalent value of dsDNA). [D] is the concentration of DNA duplex, which is changed to [DH] in analyzing the melting curve of *H-DNA*. The equilibrium fraying constant K_f can be expressed as,

$$K_f = \frac{k_o}{k_c} = \frac{[o]}{[c]} = \frac{L[D]-[c]}{[c]} \quad \text{(S4)}$$

Here, k_o and k_c are the rate constants of breaking and forming a base pair, respectively. [o] is the total concentration of broken base pairs existing in the duplex. The dissociation of DNA is a simple dimer-monomer transition thus the dissociation constant is expressed as,

$$K_d = \frac{k_d}{k_a} = \frac{[S]^2}{[D]} \quad \text{(S5)}$$

k_d and k_a are the rate constants of dissociation and association, respectively. Given the three binding partners involved, two ssDNA (*S*) and one free ligand (*H*), the formation of *H-DNA* complex can proceed in a number of possible pathways. The transition from monomers 2*S* + *H* to the ternary H-bound duplex (*DH*) can be stepwise or concerted, and two stepwise process are possible, as shown in Supplementary Fig. 5.

In the sequential association scenario, two routes are possible. One is that the formation of *D* is required for the ligand to bind, resulting in a stepwise process described by association constant K_a and binding constant K_b,

$$S + S \overset{K_a}{\rightleftharpoons} D \quad \text{(S6)}$$

$$D + H \overset{K_a}{\rightleftharpoons} DH \tag{S7}$$

In this case, K_a is easily obtained by using the parameters in Table 1. Since the total concentration of H is half of C_D ($[H]+[DH]=C_D$), the external fraction is expressed as,

$$\theta_{ext} = \theta_{DH} = \frac{[DH]}{C_D} = \frac{[D]K_b}{[D]K_b + 1} \tag{S8}$$

$$[D] = \frac{8C_D + K_a^{-1} - \sqrt{K_a^{-2} + 16C_D K_a^{-1}}}{8} \tag{S9}$$

The Gibbs free energy change associated with the binding process is

$$\Delta G_b^0(T) = -RT\ln(K_b) = \Delta H_b^0 - T\Delta S_b^0 \tag{S10}$$

As summarized in Supplementary Table 3, the obtained thermodynamic parameters are strongly dependent on C_D. As shown in Supplementary Fig. 6b, by varying C_D from 1 mM to 200 nM, K_b increasing by two order of magnitudes to 1.4×10^{7} M^{-1} , but is much smaller than the literature value 3.2×10^{8} M^{-1} measured at 25 ^{o}C,[13] indicating that this model is improper in characterizing the association process of *H-DNA*.

The second mechanism is that an interaction between the ligand and *S* facilitates the association of the second *S* to form *DH*,

$$H + S \overset{K_1}{\rightleftharpoons} SH \tag{S11}$$

$$SH + S \overset{K_2}{\rightleftharpoons} DH \tag{S12}$$

The process described by Eq. S12 refers to the situation that *H* is tightly bound to one ssDNA. In this case, the flexibility of *H* is confined, making *SH* detectable in the absorption and fluorescence measurements. Bichenkova *et al.* have shown that *H* does not bind to ssDNA in pH 7 buffer due to the presence of counter cations (Na^{+}) that disrupt the nonspecific electronic

interactions.[14] Overall, the association of *H-DNA* is not a stepwise but concerted binding process that is facilitated by the electrostatic interactions between *H* and nearby ssDNA.

5. Transition Model of *H-DNA* Reaction Rates

The formation of the *H-DNA* complex can be a complicated process due to the three binding partners involved, two ssDNA (*S*) and one free ligand (*H*). As discussed earlier, the association of H-DNA is a concerted binding process. On this basis, one can neglect *D* in describing the *DH* dissociation reaction when performed in the presence of *H*. Then, the dissociation of *H-DNA* (*DH*) into two *S* and *H* can be simply described by a two-state model,

$$DH \underset{}{\overset{K_d}{\rightleftarrows}} H + 2S \tag{S13}$$

The equilibrium dissociation constant K_d is written as,

$$K_d = \frac{k_d}{k_a} = \frac{[H][S]^2}{[DH]} = \frac{[S]^3}{2[DH]} \tag{S14}$$

Here, k_d and k_a are dissociation and association rate constants, respectively. Neglecting any *D,* the total concentration of DNA in the duplex form is $C_D = C_{tot}/2 = [DH] + [S]/2$. Assuming free *H* can only exist when the duplex is dissociated, [S] = 2[H], we can derive a relation between K_d and [S],

$$[S]^3 + K_d [S] - 2C_D K_d = 0\ . \tag{S15}$$

Since [S] has to be a real value, only one solution to Eq. S16 matches this condition,

$$[S] = \frac{K_d}{F} - \frac{F}{3} \tag{S16}$$

$$F = \sqrt[3]{a_1 - a_2} \tag{S17}$$

$$a_1 = \sqrt{27\left(27C_D^2 K_d^2 + K_d^3\right)} \tag{S18}$$

$$a_2 = 27 C_D K_d \tag{S19}$$

The obtained thermodynamic parameters are listed in Table 1, with K_d being consistent for different C_D. Moreover, we compare this model with the three-state model described by Eq. S7 and S8 earlier, by using our fluorescence titration measurement. As shown in Supplementary Fig. 7, the fluorescence spectra are plotted with log y-axis for better viewing the data quality at various C_D. In the concentration range examined (10 – 1823 nM), the fraction of *DH,* $\alpha_{DH} = [DH]/C_D$, varies from 73% to 100% as calculated with two-state model using Eq. S15. α_{DH} calculated with three-state following Eq. S7 and S8 is strongly dependent on K_b, with its value at C_D = 10 nM changing from 33% to 49% by decreasing K_b from $10^8$to 5×10^7 M^{-1}. The fluorescence intensity of *H* is enhanced dramatically upon binding to DNA, thus is proportional to [*DH*].[13] The ratio between the fluorescence counts (*F*) and [*DH*], $\varphi = F/[DH]$, should be constant. As illustrated in Supplementary Fig. 7c, φ calculated with two-state model varies slightly around 1250 in the studied C_D range. In contrast, K_b strongly affects φ which decreases significantly with increasing C_D, suggesting that the three-state model is improper in describing the association of *H-DNA*.

The mathematical treatment of these dissociation kinetics is first simplified with small-amplitude perturbation method, in which the macroscopic change in concentration is zero. It can then be derived,

$$\frac{d[DH]}{dt} = [H][S]^2 k_a - [DH] k_d \tag{S20}$$

$$\frac{d[\delta H]}{dt} = \frac{k_a}{2}\left([S]_{eq} + [s]\right)^3 - k_d\left([DH]_{eq} + [\delta H]\right) = -3[S]_{eq}^2 k_a [\delta H] - k_d [\delta H] \tag{S21}$$

where [δH] and [s] are the small changes of the equilibrium concentrations $[DH]_{eq}$ and $[S]_{eq}$, respectively. Therefore, the observed rate constant of dissociation is,

$$\lambda_d = 3[S]_{eq}^2 k_a + k_d \tag{S22}$$

Similarly, we can derive the rate constant of fraying,

$$\lambda_f = k_o + k_f \tag{S23}$$

In the dye-free solution, the formation of duplex DNA (D) is a classic monomer-dimer ($2S \rightleftarrows D$) transition. So, the expression of duplex fraction and dissociation rate are,

$$\theta_{ext} = \theta_D = 1 + \frac{\mathrm{K}_d(T)}{8C_D}\left(1 - \sqrt{1 + \frac{16C_D}{\mathrm{K}_d(T)}}\right) \tag{S24}$$

$$\lambda_d = 4[S]_{eq}\, k_a + k_d \tag{S25}$$

The calculated internal/external fractions and the association/dissociation rate constants at various temperatures are summarized in Supplementary Table 4.

6. Analysis of T-jump Kinetics

The T-jump relaxation kinetics observed in our experiments are influenced by the temperature of the buffer as it thermally re-equilibrates from T_f back to T_i. The observed time-dependence of the transient signal $S(t)$ is the convolution of the time-dependent sample response $R(t)$ and the time-dependent temperature profile $T(t)$,

$$S(t) = R(t) \otimes T(t) \tag{S26}$$

Here $T(t)$, can be characterized by the time-dependent absorbance change of IR light through a solvent sample, which is well fit to a stretched exponential of the form $\mathrm{T}(t) = \exp\left[-(\mathrm{t}/\tau_T)^{\beta}\right]$ where τ_T=13.4 ms and β=0.78. Supplementary Fig. 8a shows examples of simulated T-jump response by convolving an exponential response of the form exp[$-\lambda_{rise}$t] with $T(t)$ based on Fourier transformation.

$$S(t) = FT^{-1}\left\{FT\left[R(t)\right] \times FT\left[T(t)\right]\right\} \tag{S27}$$

To obtain the correlation between the input and observed rate, each convolved signal is fit to a bi-exponential function of the form $-C[\exp(-\lambda_{rise}\tau) - \exp(-\lambda_{decay}\tau)]$. Supplementary Fig. 8b illustrates the mapping between observed rate λ_{rise} and the underlying de-convolved rate based on our kinetic modeling. It is found that λ matches λ_{rise} when the sample response time is less than 3 ms, and our T-jump experiments fail to detect any kinetics slower than 11.6 ms.

7. Comparison of Observed Rates at Various Temperatures

The transient IR experiments display diverse relaxation phenomena spanning multiple time scales and varying detection frequencies. To assist in isolating the temporal and spectral information, we applied a maximum entropy method (MEM) analysis in the context of Laplace inversion to obtain a smooth rate distribution for the observed kinetics at each detected frequency. Our implementation of this method for analyzing transient T-jump vibrational spectra is based on the method introduced by Kumar *et al.*,[15] and has been described in detail in a recent publication.[16]

Supplementary Fig. 9 illustrates the temperature dependence of the observed dissociation rates. The rates of native DNA show a clear increase with rising temperature. *H-DNA* rates are much slower, and a fast response is centered at ~10^8 s^{-1}, but no clear temperature dependence is evident. Based on the distribution in the frequency domain of a positive rate amplitude at 1546 cm^{-1} and negative amplitudes at 1574 and 1665 cm^{-1}, this response is assigned to the dissociation of GC base pairs.

8. Arrhenius Analysis and Transition-State Theory

The linear temperature dependence of association (a) and dissociation (d) rate constants are well described by the Arrhenius equation,

$$\ln k_i = \ln A_i - E_i / RT \,, \tag{S28}$$

where E_i represents the activation energy and A_i is the pre-exponential term. Arrhenius plots of *H-DNA* and DNA are shown in Supplementary Fig. 10 and are well-described by linear fits. The absence of clear curvature in these plots indicates that there is no significant change in the activation heat capacity. Extracted parameters are listed in Supplementary Table 5. The activation free energy barriers can be estimated from the rate constants using Eyring equation,

$$k_i = k_0 \exp\left(-\Delta G_i^{\dagger}/RT\right) = k_0 \exp\left(-\Delta H_i^{\dagger}/RT\right)\exp\left(\Delta S_i^{\dagger}/R\right) \tag{S29}$$

$$k_0 = k_B T/h \tag{S30}$$

Here $\Delta S_i^{\dagger}$ and $\Delta H_i^{\dagger}$ are the activation entropy and enthalpy, respectively. According to the Eyring formalism, the attempt frequency k_0 is calculated with Planck's constant h and the Boltzmann constant k_B.

9. Transient Signal of the Hydrogen Bond Breaking between *H* and Thymine

As shown in Supplementary Fig. 11, at temperatures below 60 ^{o}C where native and *H-DNA* both form dsDNA, the differences between their IR spectra illustrate the spectral changes resulting from the ligand-induced conformational changes and local environment of DNA. The most notable feature is the sharpening and blue shift of the C^2=O thymine carbonyl stretching mode from 1698 cm^{-1} to 1702 cm^{-1}, which corresponds to the displacement of water and the binding of *H* through hydrogen-bond formation. Since the frequency of the *H* binding signal is displaced from the main dissociation spectral features, it can be used as an indicator for tracking the hydrogen bond dissociation between the *H* and thymine (T).

In dissociation kinetics, the most intuitive way to compare the time scales of the T carbonyl mode with the dissociation response is a rate distribution plot. As illustrated in Supplementary Fig. 12, the rate at maximum at 1706 cm^{-1} is larger than 1607 cm^{-1} at all temperatures, suggesting a slightly faster response resulting from *H* hydrogen-bond dissociation. Furthermore, the rising signal of the t-HDVE time trace at 1706 cm^{-1} suggests an increased response than that of 1607 cm^{-1}, although it is noisy due to its small amplitude variation with temperature change.

As shown in Supplementary Fig. 13, at 70 ^{o}C, both native and *H-DNA* are predominately dimers and their difference 2D IR spectrum primarily reflects the features correlated with the DNA conformational changes and local environment variations. The most striking feature is the loss signal centered at 1700 cm^{-1} (ω_1)/1706 cm^{-1} (ω_3), illustrated in Supplementary Fig. 13c, resulting from the breaking of the hydrogen-bonds between the ligand and DNA T carbonyls. This feature is observed in the transient difference spectrum between 2 ms and 500 ns, but absent in the spectrum at longer timescales between 4 ms and 2 ms, as shown in Supplementary Fig. 14. Given the inverse observed rate is around 2 ms and the transient signal maximizes at 4 ms, this observation is consistent with our previous speculation that hydrogen-bond dissociation is required for interrupting the strong hydrophobic interactions between the ligand and the

dsDNA minor groove. The projections of the difference t-2D IR spectra in Supplementary Fig. 14a and b onto the detection frequency axis are compared in Supplementary Fig. 14c. The loss feature of T carbonyls upon binding to *H* at 1706 cm^{-1}, is observed clearly in the projection of difference t-2D IR spectrum between 2 ms and 500 ns, but is absent in the one between 4ms and 2ms.

10. Discussion of the Association and Dissociation Processes

As illustrated in Supplementary Fig. 15, the association process of *H-DNA* is cooperative with the following sequence: (1)the ligand facilitates the diffusion process of the two ssDNA through nonspecific electrostatic interactions then (2) the critical nucleus of dsDNA forms with the help of electric attractions afforded by *H*. (3) Sliding and re-orientation of *H* proceeds simultaneously with the dsDNA formation and terminates when *H* is confined in the narrowest part of the dsDNA minor groove. The dissociation process observed with our T-jump IR experiments starts with the fraying of GC ends that have an average rate around 10^8 s^{-1}. The hydrophobic interactions between *H* and the dsDNA minor groove play an important role in stabilizing the central ATs and are strongly perturbed by the hydrogen-bond dissociation of *H* with the T carbonyls. The rate distribution of the hydrogen-bond dissociation signal is similar to the dissociation response of AT base pairs, confirming the importance of the hydrophobic interactions.

11. Convergence of the DNA Simulations

To evaluate the statistical convergence of the DNA simulations, the root-mean squared displacement (RMSD), minor groove width, helical twist, and helical propeller are calculated. The RMSD is computed with respect to the average structure of the DNA duplex over the full 5 µs simulations of DNA and *H-DNA* at three different temperatures (Supplementary Fig. 16). Using all 12 base pairs to calculate the RMSD, the RMSD exhibits jumps corresponding to fraying event (Supplementary Fig. 16a and 16b). In contrast, when the RMSD is evaluated using only the internal 8 base pairs of each trajectory (Supplementary Fig. 16c and 16d) fraying events are not observed for either DNA nor *H-DNA*.

Properties of the native dsDNA with sequence 5'-CGC AAA TTT GCG-3' are expected to be symmetric. The binding of *H* breaks this symmetry, which is especially evident in base steps 4 through 8, where direct binding occurs between *H* and the DNA (Supplementary Fig. 17). Several structural properties of the DNA and *H-DNA* systems were calculated, including the minor groove width, helical propeller, and helical twist. The calculated minor groove width values are shown in Supplementary Fig. 18. For native DNA, all the strands show symmetric distributions of the minor groove width, which indicates that the simulations are statistically converged. The simulations show a slight increase in the average minor groove width for *H-DNA*, associated with the needed space to accommodate *H* in the minor groove. As the temperature is increased in the simulations, the average minor groove width becomes slightly larger due to the increased thermal fluctuations in the DNA and water at elevated temperatures.

Another structural property to consider for convergence is the helical propeller (Supplementary Fig. 19). Again, the DNA strand shows symmetry around the A6-T7 base step indicating convergence of the simulations. Again, the *H-DNA* calculations exhibit an expected departure from the symmetry of the native DNA. A last property to consider is the average helical twist of each base step throughout the trajectory (Supplementary Fig. 20). The helical twist also shows symmetry about the A6-T7 base step for native DNA, further evidence of the convergence of the simulations. The binding of *H* once again breaks the symmetry.

12. Calculated Minor Groove Width

There are two ways to define the minor groove width. Common in crystallography is the use of the O4 – O4 distance between the selected base pair and the base pair 4 steps down the strand, for example A4 to T8, to determine the minor groove width. Utilizing this method, the minor groove width for DNA and *H-DNA* are ~ 12 Å (Supplementary Fig. 21). However, this definition is only a good measure for internal base pairs in the 12 base pair strand.

When utilizing the O4 – O4 distance metric, the A5 base step is able to capture the deviation from *H* binding because it measures the span of A5 to T9, encompassing the base pairs forming hydrogen-bonds with *H*.

Another method commonly used for calculating the minor groove width in molecular dynamics simulations is utilizing the phosphorus (P) atoms of the backbone of the DNA. Utilizing the helical twist information, vectors are defined for each P atom and a distance matrix is created from each of the P atoms. A 3D representation of the distance matrix can be created and the minima in the surface correspond to the major and minor groove widths. Full methodological discussion can be found in a paper by Lavery *et al*.[10] The latter method is used to evaluate the minor groove widths in this paper (for example, those shown in Supplementary Fig. 18). The minor groove widths and their standard deviations are tabulated in Supplementary Table 6, 7, and 8. These values are used to calculate the percentages in the main text.

13. Hydrogen-Bond Interactions in *H-DNA*

The key hydrogen-bonds between *H* and the DNA duplex are illustrated in Supplementary Fig. 22. The bonding locations are determined through hydrogen-bond analysis using a 3 Åmaximum cutoff for the bond length and a 135-180° angle range requirement. Bonds depicted in black represent the predominant hydrogen-bonds throughout the trajectory and red bonds denote transient hydrogen-bonds formed with the duplex. The transient T7* bond has an average bond length below the 3 Å cutoff but the average bond angle is not in hydrogen-bonding range, displayed in Supplementary Fig. 23 and 24. Also seen in Supplementary Fig. 23 and 24, the A6* bond has an average angle fluctuating around the cutoff value of 135° and the bond length averages below 3 Å, indicating it is the more frequent of the transient hydrogen-bonds. However, it should be noted only a single red or black bond is presented at any given time because the NH bonds of the *H* molecule are only capable of donating a single hydrogen-bond.

Supplementary Methods

Sample Preparation: DNA sequences AAA TTT (A_3T_3), CGC GCG ($(CG)_3$) were purchased from Integrated DNA Technologies (IDT) at desalt-grade purity. Oligonucleotides were further purified using membrane tubing with a 0.5 kD MWCO (Spectrum Micro Float-A-Lyzer) to perform dialysis against distilled water at 0 ^{o}C for 8 hours. Deuterated buffer with 10 mM Tris and 200 mM NaCl at pH 7.4 is used in the study of A_3T_3 and $(CG)_3$. An additional 50 mM $MgCl_2$ is used for stabilizing the A_3T_3 duplex. Buffer pH's were adjusted with 1 M HCl and NaOH solutions then checked with a pH meter (Fisherbrand accumet AB150). The concentration of oligonucleotide and *H* was checked on a NanoDrop UV/vis spectrometer (Thermo Scientific).The concentration of native and *H-DNA* refers to the equivalent value of double-stranded polymers. Prior to measurements, all samples were annealed by heating to 95 °C for 3 min and then cooled gradually to room temperature over 10–15 min. For the IR measurements, labile protons of DNA and *H* were HD exchanged in deuterated water (D_2O, Cambridge Isotopes) and lyophilized before dissolving into the deuterated buffer.

Fluorescence Titration: The fluorescence titration experiments were carried out using Horiba Fluorolog-3 at 1 nm resolution. The samples were excited at 345 nm. Emission spectra in the range of 365-650 nm were recorded. A quartz cuvette with 0.5 cm path length is used. For each titration, 1 µL *H-DNA* solution was added with concentration varied from 5 µM to 50 µM via serial dilution from the 100 µM stock solution.

Molecular Dynamics Simulations: The DNA sequence 5'-CGC AAA TTT GCG-3' is used for all molecular dynamics (MD) simulations, which are carried out using Amber 18.0[1] with the bsc1 force field for DNA[2] and SPC/E water model.[3] The Na^+ and Cl^- ions are treated with the force field by Joung and Chetham,[4,5] and the ligand was previously parameterized by Furse *et al*.[6] Initial structures for DNA and *H-DNA* are obtained from their crystal structures, 1S2R[7] and 264D[8] respectively. The starting structures are solvated in a periodic rectangular box that allows at least 10 Å of buffer between any DNA or solute atom to the edge of the box. 250 mM NaCl was added in addition to the neutralizing Na^+ ions for each system. The details of each system are listed in Supplementary Table 1.

Two energy minimizations are performed on the full system to allow the solvent to relax and to remove any steric hinderances in the DNA from the crystal structure. First, 500 steps of steepest decent are followed by 500 conjugate gradient steps with a 500 kcal mol^{-1} Å^{-2} constraint on the DNA or *H-DNA* to allow solvent relaxation. All constraints are removed and an additional 1000 steps of steepest decent followed by 1500 steps of conjugate gradient to allow the DNA to relax. The system is then heated from 0 K to the desired temperature of 300 K, 333 K, or 343 K while the DNA or *H-DNA* is harmonically restrained by a 25 kcal mol^{-1} Å^{-2} force constant, and the solvent and ions can move freely. The temperature is regulated with a Langevin temperature control and the volume is held constant (NVT) during the 100 ps heating simulation. To maintain the structural integrity of the DNA while allowing the solvent to relax to achieve a proper density, the restraints are released in 125 ps simulations at a constant pressure and temperature (NPT). The restraints are released by 5 kcal mol^{-1} Å^{-2} at a time over 25 ps intervals from 25 kcal mol^{-1} Å^{-2} to 5 kcal mol^{-1} Å^{-2}. Weak coupling to a Berendsen piston is used to maintain a pressure of 1 bar. Once all restraints have been released from the DNA, an additional 100 ps of NPT simulation is performed to allow the entire system to reach the proper density. To create a box with the appropriate density, each side of the box is multiplied by a scalar factor, *d*,

$$d = \left(V'/V\right)^{\frac{1}{3}} \qquad \text{(S31)}$$

where *V'* is the average volume of the box for the duration of the 100 ps simulation and *V* is the volume of the box at the last frame of the 100 ps simulation. After the box has been properly adjusted for the correct density, a 1 ns simulation is performed to allow the system to relax in the NVT ensemble before production runs. During the MD simulations, the long-ranged electrostatics are treated with the particle-mesh Ewald method using a real-space cut-off of 9.0 Å. The SHAKE algorithm is employed to constrain all bonds containing hydrogen, which then allows for a 2 fs timestep. Analysis is performed on a 5 μs production simulation utilizing the Cpptraj[9] and Curves+[10] analysis programs.

Supplementary References

1 D.A. Case, I. Y. B.-S., S.R. Brozell, D.S. Cerutti, T.E. Cheatham, III, V.W.D. Cruzeiro, T.A. Darden, R.E. Duke, D. Ghoreishi, M.K. Gilson, H. Gohlke, A.W. Goetz, D. Greene, R Harris, N. Homeyer, Y. Huang, S. Izadi, A. Kovalenko, T. Kurtzman, T.S. Lee, S. LeGrand, P. Li, C. Lin, J. Liu, T. Luchko, R. Luo, D.J. Mermelstein, K.M. Merz, Y. Miao, G. Monard, C. Nguyen, H. Nguyen, I. Omelyan, A. Onufriev, F. Pan, R. Qi, D.R. Roe, A. Roitberg, C3. Sagui, S. Schott-Verdugo, J. Shen, C.L. Simmerling, J. Smith, R. SalomonFerrer, J. Swails, R.C. Walker, J. Wang, H. Wei, R.M. Wolf, X. Wu, L. Xiao, D.M. York and P.A. Kollman AMBER 2018. *University of California, San Francisco* (2018).

2 Ivani, I. *et al.* Parmbsc1: a refined force field for DNA simulations. *Nat. Methods* **13**, 55-58 (2016).

3 Berendsen, H. J. C., Grigera, J. R. & Straatsma, T. P. The Missing Term in Effective Pair Potentials. *J. Phys. Chem.* **91**, 6269-6271 (1987).

4 Joung, I. S. & Cheatham, T. E. Determination of alkali and halide monovalent ion parameters for use in explicitly solvated biomolecular simulations. *J. Phys. Chem. B* **112**, 9020-9041 (2008).

5 Joung, I. S. & Cheatham, T. E. Molecular Dynamics Simulations of the Dynamic and Energetic Properties of Alkali and Halide Ions Using Water-Model-Specific Ion Parameters. *J. Phys. Chem. B* **113**, 13279-13290 (2009).

6 Furse, K. E. & Corcelli, S. A. The dynamics of water at DNA interfaces: Computational studies of Hoechst 33258 bound to DNA. *J. Am. Chem. Soc.* **130**, 13103-13109 (2008).

7 Woods, K. K. *et al.* High-resolution structure of an extended A-tract: [d(CGCAAATTTGCG)]2. *J. Am. Chem. Soc.* **126**, 15330-15331 (2004).

8 Vega, M. C. *et al.* Three-dimensional crystal structure of the A-tract DNA dodecamer d(CGCAAATTTGCG) complexed with the minor-groove-binding drug Hoechst 33258. *Eur. J. Biochem.* **222**, 721-726 (1994).

9 Roe, D. R. & Cheatham, T. E. PTRAJ and CPPTRAJ: Software for Processing and Analysis of Molecular Dynamics Trajectory Data. *J. Chem. Theory Comput.* **9**, 3084-3095 (2013).

10 Lavery, R., Moakher, M., Maddocks, J. H., Petkeviciute, D. & Zakrzewska, K. Conformational analysis of nucleic acids revisited: Curves. *Nucleic Acids Res.* **37**, 5917-5929 (2009).

11 Ramakers, L. A. *et al.* 2D-IR Spectroscopy Shows that Optimized DNA Minor Groove Binding of Hoechst33258 Follows an Induced Fit Model. *J. Phys. Chem. B* **121**, 1295-1303 (2017).

12 Ashwood, B., Sanstead, P. J., Dai, Q., He, C. & Tokmakoff, A. 5-Carboxylcytosine and Cytosine Protonation Distinctly Alter the Stability and Dehybridization Dynamics of the DNA Duplex. *J. Phys. Chem. B* (2020).

13 Haq, I., Ladbury, J. E., Chowdhry, B. Z., Jenkins, T. C. & Chaires, J. B. Specific binding of hoechst 33258 to the d(CGCAAATTTGCG)2 duplex: calorimetric and spectroscopic studies. *J. Mol. Biol.* **271**, 244-257 (1997).

14 Bichenkova, E. V., Frau, S., Fedorova, O. S. & Douglas, K. T. Binding of a desmetallo-porphyrin conjugate of Hoechst 33258 to DNA. III. Strong binding to single-strand oligonucleotides. *Nucleos. Nucleot. Nucl.* **20**, 157-168 (2001).

15 Kumar, A. T. N., Zhu, L. Y., Christian, J. F., Demidov, A. A. & Champion, P. M. On the rate distribution analysis of kinetic data using the maximum entropy method: Applications to myoglobin relaxation on the nanosecond and femtosecond timescales. *J. Phys. Chem. B* **105**, 7847-7856 (2001).

16 Sanstead, P. J. & Tokmakoff, A. Direct Observation of Activated Kinetics and Downhill Dynamics in DNA Dehybridization. *J. Phys. Chem. B* **122**, 3088-3100 (2018).
